# NEK5 interacts with LonP1 and its kinase activity is essential for the regulation of mitochondrial functions and mtDNA maintenance

**DOI:** 10.1002/2211-5463.13108

**Published:** 2021-02-24

**Authors:** Camila de Castro Ferezin, Fernanda Luisa Basei, Talita D. Melo‐Hanchuk, Ana Luisa de Oliveira, Andressa Peres de Oliveira, Mateus P. Mori, Nadja C. de Souza‐Pinto, Jörg Kobarg

**Affiliations:** ^1^ Faculdade de Ciências Farmacêuticas Universidade Estadual de Campinas Brazil; ^2^ Instituto de Biologia Departamento de Bioquímica e Biologia Tecidual Universidade Estadual de Campinas Brazil; ^3^ Departamento de Bioquímica Instituto de Química Universidade de São Paulo Brazil

**Keywords:** LonP1, mitochondria, mtDNA, NEK kinases, NEK5, TFAM

## Abstract

Little is known about Nima‐related kinase (NEKs), a widely conserved family of kinases that have key roles in cell‐cycle progression. Nevertheless, it is now clear that multiple NEK family members act in networks, not only to regulate specific events of mitosis, but also to regulate metabolic events independently of the cell cycle. NEK5 was shown to act in centrosome disjunction, caspase‐3 regulation, myogenesis, and mitochondrial respiration. Here, we demonstrate that NEK5 interacts with LonP1, an AAA+ mitochondrial protease implicated in protein quality control and mtDNA remodeling, within the mitochondria and it might be involved in the LonP1‐TFAM signaling module. Moreover, we demonstrate that NEK5 kinase activity is required for maintaining mitochondrial mass and functionality and mtDNA integrity after oxidative damage. Taken together, these results show a new role of NEK5 in the regulation of mitochondrial homeostasis and mtDNA maintenance, possibly due to its interaction with key mitochondrial proteins, such as LonP1.

## Graphical abstract

mtDNA mutations have been increasingly observed in human cancers. Cumulative mtDNA damage declines cell and tissue function and underpins the aging process. Protein kinases selectively modify proteins and a plethora of signaling pathways, leading to a specific physiological responses. Here, we demonstrate how NEK5 kinase can contribute to the maintenance and repair of mtDNA.

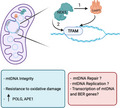

**Abbreviations**
BER: base excision repair
CCCP: carbonyl cyanide *m*‐chlorophenyl hydrazone
CS: citrate synthase
DDR: DNA damage response
IP: immunoprecipitation
IP‐LC‐MS/MS: immunoprecipitation followed by liquid chromatography coupled to mass spectrometry analysis
mtBER: mitochondrial base excision repair
mtROS: mitochondrial reactive oxygen species
NEK: Nima‐related kinase
OXPHOS: oxidative phosphorylation
PLA: proximity ligation assay
qPCR: quantitative PCR
ROS: reactive oxygen species
TMRE: tetramethylrhodamine
ΔΨm: mitochondrial membrane potential

The Nima‐related kinases (NEKs) are a family of eleven widely conserved kinases [1, 2] that have multiple mitotic and nonmitotic functions, but still much remain to be uncovered [3]. NEKs may be assigned to three major functional contexts: centrioles and mitotic spindle functions (NEK2, NEK5, NEK6, NEK7, and NEK9), primary ciliary function (NEK1, NEK4, NEK8, and NEK10), and G2/M phase‐associated DDR (NEK1, NEK5, NEK4, NEK6, NEK8, NEK10, and NEK11) [1, 2, 4, 5, 6]. Recently, many publications have been addressing new roles of NEK family members apart from cell‐cycle control [3], including regulation of cytosolic proteins [7, 8], mitochondrial function and regulation of apoptosis [9, 10, 11, 12], autophagy [13, 14], inflammasome response [15, 16], and splicing [17, 18], apart from their involvement in disease processes [19, 20].

NEK5 is one of the least characterized members of the NEK family, both structurally and functionally (Fig. 1A) and, as other NEK family members, was shown to be involved in the regulation of centrosome integrity leading to delayed centrosome separation, reduced microtubule nucleation, and errors in chromosome segregation upon its depletion [1, 21]. NEK5 was also demonstrated to be a substrate of caspase‐3 and to promote myogenesis through the upregulation of caspase‐3 activity [9]. Our research group showed that NEK5 is also located in mitochondria, where it interacts with local proteins, such as COX11 and MTX2, and its knockdown leads to altered mitochondrial respiration and increase in ROS production [8]. Recently, we also demonstrated that NEK5 participates in nuclear DNA damage response (DDR) and interacts with topoisomerase IIβ [4], one of the only topoisomerase isoforms present in mtDNA [22].

**Fig. 1:**
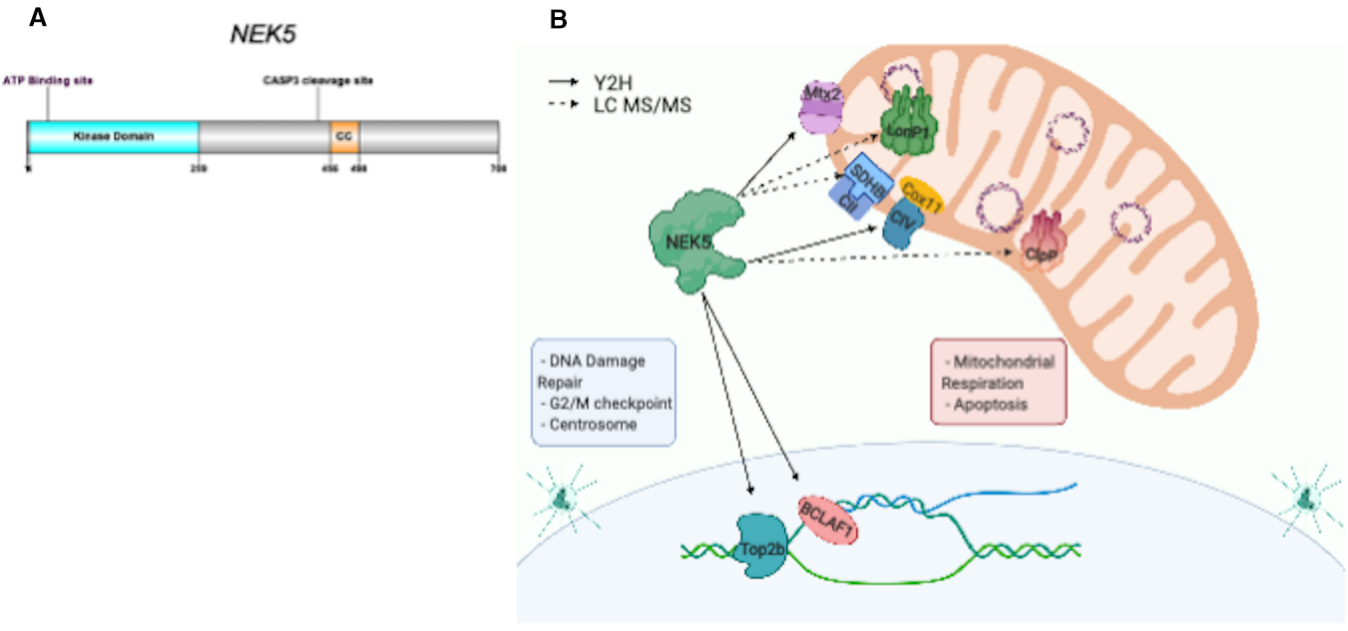
(A) NEK5 linear sequence representation: Kinase domain is localized in the N‐terminal of NEK5 kinase, with ATP‐binding site located at Lys 33. Caspase‐3 cleavage site is located between amino acids 456 and 498 (see Ref. [9]). Still, information about NEK5 function and structure remains unknown. (B) NEK5 nuclear and mitochondrial interactors. Representative illustration showing NEK5 interactors. Dotted lines represent the putative NEK5 partners identified by IP‐LC‐MS/MS, and full lines represent already published interactors identified by yeast two‐hybrid (Y2H). NEK5 has both nuclear and mitochondrial partners, and we still lack information if NEK5 exerts independent roles or if its functions are associated with a nuclear–mitochondrial communication network.

The human mtDNA is a double‐stranded circular structure containing 37 genes, which encodes essential components of the oxidative phosphorylation system (OXPHOS), and components of the mitochondrial translation system, transfer, and ribosomal RNA molecules [23]. A typical mammalian cell harbors between 1000 and 10 000 copies of the mtDNA genome that exist in a DNA–protein complex, termed nucleoid. The compaction of mtDNA is attributed to TFAM (transcription factor A, mitochondrial), that specifically binds along the mtDNA, instigating bending and looping, resulting in a nucleoid tightly compacted. The stable protein filaments that are formed between TFAM and mtDNA prevent POLRMT—mtDNA RNA polymerase—and TWINKLE—mtDNA helicase—from accessing the mitochondrial genome [23]. LonP1, an AAA+ mitochondrial protease, specifically degrades phosphorylated TFAM, leading to mtDNA unwind, influencing mtDNA transcription and nucleoid dynamic [24].

Given the indispensable role of mitochondrially encoded proteins in regulating energy production in the cell, enzymes responsible for maintaining mtDNA stability and integrity are of critical importance to cellular energetics. The maintenance of mtDNA integrity is complicated by the fact that mtDNA is particularly vulnerable to damage, especially through the generation of oxidative lesions, due to its close proximity to the site of reactive oxygen species (ROS) production in the mitochondrial membrane [25].

Due to the fact that NEK5 interacts with mitochondrial partners and participates in mitochondrial metabolism, here, we explored NEK5 mitochondrial interacting network, which led us to investigate its potential role in mtDNA maintenance and other mitochondria‐related functions.

## Materials and methods

### Cell culture, cell transfection, and chemicals

HEK293T and U2OS cells were obtained from ATCC. Flp‐In™ T‐Rex™ 293T cell line was obtained from Invitrogen™. NEK5 knockdown and NEK5 Flp‐In cell line generation have already been described elsewhere (Melo‐Hanchuk *et al*., 2015). Cells were maintained in a humid incubator with 5% CO_2_ at 37 °C and cultivated in high‐glucose Dulbecco's modified Eagle's medium (Gibco Thermo Fisher Scientific, Waltham, MA, USA) supplemented with 10% certified FBS (Gibco™). Hygromycin B was used for selection and maintenance of Flp‐In™ T‐REx™ 293T cell lines at 50 μg·mL^−1^ (Gibco™). Flp‐In™ T‐REx™ 293T cells were induced with tetracycline hydrochloride (Thermo Fisher Scientific) at 2 μg·mL^−1^ for 48 h prior to the experiments. For mtDNA damage assays, cells were incubated with zeocin (Invitrogen, Thermo Fisher Scientific) at 300 μg·mL^−1^ for 3 h and rotenone (R8875; Sigma‐Aldrich, San Luis, MO, USA) at 2 μm for 1 h. For LonP1 overexpression, pcDNA3.2‐LonP1‐flag and control vector (pcDNA5.1‐flag empty vector) were transfected Lipofectamine 2000 Transfection Reagent (Thermo Fisher Scientific), following the manufacturer's recommended protocol, for 48 h prior to the experiments. For co‐transfection followed by Co‐IP, pBABE‐MYC‐NEK5^WT^‐BioID (expected molecular weight ~ 125 kDa) and pcDNA3.2LonP1‐flag were used.

### Antibodies and fluorescent dyes

The following primary antibodies were used in immunoblotting: rabbit anti‐NEK5 (Atlas Antibodies®, HPA, HPA035565, 1 : 1000); rabbit anti‐LonP1 (Sigma‐Aldrich, Atlas Antibodies®, HPA, HPA002034); mouse anti‐Nek5 antibody (Santa Cruz Biotechnology, Santa Cruz, CA, USA, G‐12, sc‐515457); mouse anti‐GAPDH (Santa Cruz Biotechnology, FL‐335, sc‐25778, 1 : 1000); goat anti‐TFAM (Santa Cruz Biotechnology, E‐16, sc‐30963‐1 : 500); mouse anti‐tubulin (Santa Cruz Biotechnology, Tu‐02, sc‐8035, 1 : 1000); mouse anti‐Tom20 (BD Biosciences, Franklin Lakes, NJ, USA, 612278, 1 : 1000); mouse anti‐FLAG M2 (Merck, 1 : 5000); and Myc‐Tag (9B11) mouse mAb (Cell Signalling Technology, Danvers, MA, USA #2276 1 : 5000).

For immunoprecipitation (IP), anti‐FLAG® M2 Affinity Gel (Millipore, Billerica, MA, USA A2220) was used.

For immunofluorescence, rabbit anti‐LonP1 (Atlas Antibodies®, HPA, HPA002034, 1 : 100); mouse anti‐Nek5 antibody (Santa Cruz Biotechnology, G‐12, sc‐515457, 1 : 100); and Alexa Fluor‐conjugated secondary antibodies were used at 1 : 500 dilution: anti‐goat Alexa Fluor 647; anti‐mouse Alexa Fluor 488; and anti‐goat Alexa Fluor 488. MitoTracker™ Deep Red FM (Invitrogen, M22426) was used for mitochondrial visualization.

### Immunoprecipitation

Flp‐In™ T‐REx™ 293T expressing pcDNA5.1 empty vector and Flp‐In™ T‐REx™ 293T expressing pcDNA5.1 flag‐NEK5 *wild‐type* (NEK5^WT^) or pcDNA5.1 flag‐NEK5 K33A (NEK5^K33A^) were induced with tetracycline for 48 h. Cells were lysed with lysis buffer (50 mm Tris 7,4, 100 mm NaCl, 1 mm DTT, 1 mm EDTA, 30 μg·mL^−1^ DNase I, 1% Triton X‐100) supplemented with protease and phosphatase inhibitor cocktail (Roche Applied Science, Mannheim, Germany). Flp‐In™ T‐REx™ 293T Flag and Flag‐NEK5 lysates were incubated with anti‐FLAG M2 Agarose Affinity Gel (Sigma‐Aldrich) during 3 h at 4 °C. Immunoprecipitated (IP) complexes using anti‐FLAG were eluted twice with 3× FLAG peptide for 30 min (IP). The lysates and IP samples were resolved in SDS/PAGE gels and immunoblotted using anti‐FLAG antibody and rabbit anti‐LonP1 (Atlas Antibodies®, HPA, HPA002034, 1 : 1000). For reverse‐IP experiment, HEK293T cells were co‐transfected with pBABE‐MYC‐NEK5^WT^ and pcDNA3.2LonP1‐flag, or pBABE‐MYC‐NEK5^WT^ and pcDNA3.2 empty vector (control) using the transfection protocol described previously at Cell culture, cell transfection and chemicals. After 48 h, lysates were harvested and incubated with anti‐FLAG M2 Agarose Affinity Gel (Sigma‐Aldrich) during 1 h at 4 °C. IP complexes were eluted with 2× Sample Loading Buffer and boiled for 10 min. The lysates and IP samples were resolved in SDS/PAGE gels and immunoblotted using anti‐FLAG antibody, rabbit anti‐LonP1 (Atlas Antibodies®, HPA, HPA002034, 1 : 1000), anti‐Myc‐Tag (9B11), mouse mAb (Cell Signalling, #2276 1 : 5000), and rabbit anti‐NEK5 (Atlas Antibodies®, HPA HPA035565, 1 : 1000).

### Mass spectrometry (IP‐LC‐MS/MS)

For proteomic analysis, about 100 μg protein of IP (Immunoprecipitation) complexes was reduced in 500 μm dithiothreitol for 30 min at 56 °C, alkylated with 4 mm iodoacetamide for 30 min at room temperature (RT) protected from light, and digested with 20 ng·μL^−1^ trypsin (Promega, Madison, WI, USA). The samples were precleared through a Sep‐Pak Column (Merck‐Millipore) to avoid contaminants. Thus, digested peptides were dried in a vacuum concentrator and reconstituted in 50 μL of 0.1% formic acid and 5 μL of the suspension was analyzed in a ETD‐enabled LTQ Velos Orbitrap mass spectrometer (Thermo Fisher Scientific) coupled to LC‐MS/MS by an EASY‐nLC System (Proxeon Biosystems, Roskilde, Denmark) through a Proxeon nanoelectrospray ion source or an RP nano‐UPLC (nanoACQUITY; Waters, Milford, MA, USA) coupled with a Q‐Tof Ultima mass spectrometer (Waters). All of the instrument methods for the LTQ Velos Orbitrap were set up in the data‐dependent acquisition mode. Peak lists (msf) were generated from the raw data files using Proteome Discoverer version 1.3 (Thermo Fisher Scientific) with SEQUEST search engine against Swiss‐Prot human database with carbamidomethylation (+57.021 Da) as the fixed modification and methionine oxidation (+15.995 Da) as variable modification, allowing one trypsin missed cleavage site and a tolerance of 10 p.p.m. for precursor and 1 Da for fragment ions. The msf files generated by Proteome Discoverer software were analyzed in Scaffold Q+ v.3.3.2 (proteome Software, Portland, OR, USA), with scoring parameters adjusted to obtain a false discovery rate of < 1%. The peptides with a minimum of five amino acid residues and showing a significant threshold (*P* < 0.05) in Mascot‐based scores were considered in the data. The peptides identified in the mass spectrometry were considered only if (a) IP in at least three replicates of FLAG‐NEK5 samples and (b) detected in none or only one replicate of control‐FLAG IP. The LC‐MS/MS assays were performed at the Mass Spectrometry Facility in the Brazilian Bioscience National Laboratory (LNBio‐CNPEM, Campinas, SP, Brazil).

### In silico protein–protein interaction network analysis

The proteomic data retrieved from IP‐LC‐MS/MS were submitted to the Integrated Interactome System (IIS) platform (National Laboratory of Biosciences, Campinas, Brazil) [26]. The enriched biological processes from the Gene Ontology (GO) database were calculated using the hypergeometric distribution [27]. The protein network was assembled using cytoscape 3.7.0 software [28]. The prediction of phosphorylated sites was performed using Scansite 4.0 [29]. For enriched pathway analysis, Metascape (https://metascape.org/gp/index.html#/main/) was used.

### Isolation of crude mitochondria

Flp‐In™ T‐REx™ 293T Flag and Flag‐NEK5^WT^ cells were rinsed with 5 mL PBS, trypsinized, and centrifuged at 250 ***g*** for 5 min. Cell pellets were resuspended in 4.5 mL of 1× IB‐1 buffer (225 mm mannitol, 75 mm sucrose, 0.1 mm EGTA, 30 mm Tris/HCl pH 7.4) and homogenized with ~ 100 strokes in a glass homogenizer, manually. The homogenate (whole‐cell extract) was centrifuged for 10 min at 800 ***g*** and the pellet discarded, twice. Approximately 100 μL of the supernatant (PNS) was harvested for immunoblotting analyses. The remaining PNS was centrifuged at 10 000 ***g*** for 20 min, and the resulting pellet (MITO) and the cytosolic fraction (CYT) were separated for two different procedures. The MITO fraction was washed with ice‐cold IB‐1 buffer and centrifuged at 8500 ***g*** for 10 min. The pellet was resuspended in 1× IB‐2 buffer (225 mm mannitol, 75 mm sucrose, 30 mm Tris/‐HCl pH 7.4), followed by centrifugation at 10 000 ***g*** for 10 min, twice. After centrifugation, the pellet was resuspended in 800 μL of mitochondrial resuspending buffer (250 mm mannitol, 5 mm HEPES pH7.2, 0.5 mm EGTA). A 1 mL aliquot of CYT was ultracentrifuged at 34 000 ***g*** for 1 h at 4 °C (Optima L‐90K Beckman‐Coulter Rotor: sw −41Ti speed: 28 500 r.p.m.) (adapted protocol [30]).

### Immunofluorescence and confocal assays

For NEK5 co‐localization experiments, U2OS cells were grown on coverslips and, when necessary, stained with MitoTracker Deep Red (200 nm) for 30 min, and subsequently fixed and permeabilized with ice‐cold methanol for 15 min at −20 °C. Fixed cells were rehydrated in PBS for 10 min, blocked in PBS‐T (1× PBS, 0.1% Triton X‐100, and 3% BSA) for 20 min, and then incubated in the same buffer with the primary antibodies described previously. After three washes with PBS‐T, cells were incubated with secondary antibodies in the same buffer for 30 min. After this incubation, slides were rinsed with PBS‐T, and the coverslips were mounted on a microscope slide with ProLong Antifade Reagent. The confocal acquisitions were performed at the National Institute of Science and Technology on Photonics Applied to Cell Biology (INFABIC) with a confocal LSM 510 microscope and treated using fiji software (Tokyo, Japan) [31]. For colocalization analyses, images were processed with background correction using 50.0 pixels in a ball rolling radius and applying median filtering. Fiji Coloc2 plugin was used for Pearsons’ correlation coefficient calculation.

### Immunoblot

Whole‐cell lysates, and cytosolic or mitochondrial fractions were used in western blotting, with independent experiments repeated three times. For the extraction of total cellular protein, cells were washed with PBS and lysed with lysis buffer [50 mm Tris/HCl (pH 7.4), 150 mm NaCl, and 1 mm EDTA (pH 7.4), 1% NP‐40, protease and phosphatase inhibitor cocktail] for 30 min followed by centrifugation at 20 000 ***g*** for 15 min. Protein concentrations were estimated by Pierce BCA protein assay (Thermo Fisher Scientific). The whole‐cell lysate was mixed with a 1/5 volume of 5 × SDS sample buffer and boiled. Total cell proteins were resolved by SDS/PAGE followed by electrotransfer of proteins onto a poly(vinylidene difluoride) membrane. The membrane was blocked for 1 h at RT using 0.5% BSA/Tris‐buffered saline with Tween (TBST). The membrane was then incubated with antibodies listed above (Antibodies and Fluorescent dyes) overnight at 4 °C. After washing with TBST three times for 5 min, the membrane was incubated with horseradish peroxidase‐conjugated anti‐rabbit IgG at 1 : 5000 and anti‐mouse IgG at 1 : 2000 (Santa Cruz Biotechnology) for 1 h at RT. After washing with TBST three times for 5 min, protein bands were detected with peroxidase‐conjugated antibodies and blots were developed using the enhanced chemiluminescence ECL Western Blotting System (Amersham, Chalfont St. Giles, UK).

### Relative mitochondrial DNA copy number

Relative mtDNA content was determined as described in Rooney *et al*. (2015). Total cellular DNA was isolated using the DNeasy Mini Kit (Qiagen, Hilden, Germany), and DNA concentrations were determined spectrophotometrically with NanoDrop. Equal amounts of total DNA were assayed by quantitative PCR (qPCR) with SYBR Green Mix (Applied Biosystems, Thermo Fisher Scientific). Samples were analyzed at least in triplicates, using two independently isolated DNA samples in a CFX384 Touch™ Real‐Time PCR Detection System (Bio‐Rad). The comparative Ct method was applied for quantification of mtDNA copy number, comparing the amplification of ND1 (mitochondrial gene) to that of B2 microglobulin (nuclear gene). Primer pair sequences are in presented in Table 1.

**Table 1:** Oligonucleotide sequence.

GENE	Forward (5'‐3')	Reverse (5'‐3')
OGG1	GATGTTACCCTGGCTCAA	GATGTTGTTGTTGGAGGAA
POLG	AAGCACTGTCTCGAACAGGG	CACTGCAGCTCGCAAGTTCT
APE1	TGGAATGTGGATGGGCTTCGAGCC	AAGGAGCTGACCAGTATTGATGA
TWINKLE	AGTCGTCGAGATGCTGAGGT	CATCGTTTGGGGTTCAGTTT
xL‐mito	TGAGGCCAAATATCATTCTGAGGGGC	TTTCATCATGCGGAGATGTTGGATGG
GAPDH	CGGAGTCAACGGATTTGGTCGTAT	ATGGACTGTGGTCATGAGTCCTTC
ACTB	CTCCTTAATGTCACGCACGAT	CATGTACGTTGCTATCCAGGC
ND1	ACTACGCAAAGGCCCCAACG	GAGCTAAGGTCGGGGCGGTG
B2 microglobulin	TGCTGTCTCCATGTTTGATGTATCT	TCTCTGCTCCCCACCTCTAAGT
LONP1	GTTCCCGCGCTTTATCAAGAT	GTAGATTTCATCCAGGCTCTC

### Flow cytometry

For mitochondrial oxidant measurement and mitochondrial membrane potential (ΔΨm), Flp‐In™ T‐REx™ 293T Flag and Flag‐NEK5^WT^ cells were plated in 12‐well plates 72 h before for protein expression induced by tetracycline. On the day of the experiment, probes were added to complete culture medium. MitoSOX Red (Thermo Scientific, # M36008) was added at 5 µm for 30 min and was simultaneously incubated with 0, 0.5, 1, or 2 µm of antimycin A. For ΔΨm detection, tetramethylrhodamine, ethyl ester, and perchlorate (TMRE) (Thermo Fisher Scientific, T669) were utilized at a final concentration of 200 nm, with carbonyl cyanide m‐chlorophenyl hydrazone (CCCP; C2759; Sigma‐Aldrich) at a final concentration of 10 μm used as positive control for 30 min. After the incubation, cells were washed once with PBS, trypsinized, and resuspended in 500 μL of PBS containing 2% FBS. The analysis was performed immediately in a BD FACS Verse. Median fluorescence intensity (MFI) from nonstained samples was used to normalize each cell, and normalized MFI from control, Flp‐In™ T‐REx™ 293T Flag cells, was set as 100%. The graph represents the relative fluorescence of the NEK5^WT^cells to control.

### Citrate synthase activity assay

Citrate synthase (CS) activity was evaluated spectrophotometrically as described in Hansford and Castro [32]. For this experiment, the following solutions were prepared: Tris/HCl 0.1 m, pH 8.1; 5 mm acetyl CoA, 10 mm DTNB, and 25 mm oxaloacetate diluted in Tris/HCl 0.1 m, pH 8.1; and 10% Triton X‐100 (w/v). The reaction, with a final volume of 200 μL, consisted of 0.1 m Tris/HCl, pH 8.1, 50 μm acetyl/CoA, 100 μm DTNB, 250 μm oxaloacetate, and Triton X‐100 0.1% (w/v). The reaction was initiated by adding 2 μL of protein cell extract to the concentration of 3 mg·mL^−1^ (6 μg protein per well). CS activity was spectrophotometrically measured in a Eon™ Microplate Spectrophotometer—BioTek at a wavelength of 412 nm on a reading basis every 19 s (15 s for acquisition, 3 s for shaking, and 1 s for hold) for 5 min at 30 °C. Enzyme activity was estimated through the formation of the reaction‐derived DNTB‐CoA, and the value was calculated from the NBT molar extinction coefficient (ε = 13.6 ∙ 10^−6^ m ^−1^ ∙ cm^−1^ or 13.6 μm ∙ cm^‐1^) and adjusted to represent the number of moles of citrate formed by minute per milligram of protein (nmol·min^−1^·mg^−1^).

### Long‐extension PCR

Total DNA from Flp‐In™ T‐REx™ 293 Flag, Flag‐NEK5^WT^, and Flag NEK5^K33A^ cells was extracted using DNeasy Blood and Tissue Kit (Qiagen) following the specification of the manufacturer. The amplification of long mitochondrial fragment was performed using AccuPrime Taq DNA Polymerase High Fidelity Kit (Invitrogen™). The mitochondrial fragment (16 540 bp) was amplified using the oligos *xL‐mito* (Table S1). Densitometry was used to measure the intensity of the bands with fiji software. The quantification of the long fragment was normalized to amplification of a short (210 nt) fragment, the ND1 amplicon, to normalize for sample variations in DNA quantities.

### Analysis of gene expression

Total RNA was extracted using TRIzol Reagent (Invitogen™) following the manufacturer's instructions. The expression levels of POLγ, OGG1, APE1, and TWINKLE mRNA were measured by quantitative RT–qPCR assays, with primers targeting the mRNAs codified by these genes, and β‐actin (*ACTB*) as loading control. GAPDH was also used as a housekeeping gene and compared to *ACTB* expression, which showed no significant difference (not shown). Total RNA extracted from Flp‐In™ T‐REx™ 293 Flag, Flag‐NEK5, and Flag NEK5 K33A cells was used to generate cDNA with SuperScript™ II Reverse Transcriptase (Invitrogen™). Quantitative RT–PCRs were performed using the Power SYBR Green PCR Master Mix (Applied Biosystems), according to directions, using 15 ng template and 200 nm of each primer. The assay was performed in a CFX384 Touch™ Real‐Time PCR Detection System (Bio‐Rad), and the instrument software was used for the subsequent analysis. The oligonucleotide sequences are presented below (Table 1).

### Proximity ligation assay (PLA)

U2OS cells were cultivated in a 384‐well cell carrier plate (PerkinElmer Inc.; Waltham, MA, USA). The cells were fixed with 3.7% formaldehyde for 20 min and then permeabilized with 0.1% Triton X‐100 in 1× PBS. Cells were incubated first with Duolink® blocking solution (Sigma) for 30 min and then with primary antibodies: anti‐NEK5 (8 μg·mL^−1^; Santa Cruz Biotechnologies), anti‐TFAM (8 μg·mL^−1^; Santa Cruz Biotechnologies), and anti‐LonP1 (8 μg·mL^−1^ Atlas Antibodies®, HPA, HPA002034) diluted in Duolink® Antibody Diluent (Sigma) for 2 h, at 37 °C in humidity chamber. Next, the cells were submitted to the PLA protocol according to the manufacturer's instructions. Briefly, the cells were incubated with secondary antibodies, anti‐mouse plus, and anti‐rabbit minus, conjugated to oligonucleotides, which anneal with their complementary regions only when the two different antibodies are in close proximity (< 40 nm). The cells were then submitted to a ‘rolling‐circle’ amplification reaction and hybridized to add far‐red fluorescent probes labeled oligonucleotides, to visualize the proximity‐dependent signal. Nuclei were counterstained with Hoechst 33342 dye. Cell visualization and image data collection were using Leica (Wetzlar, Germany) DMIL LED FLUO microscope at 20×/40 CORR PH1 objective and filters corresponding to DAPI and Y5. Spots representing NEK5 and TFAM or NEK5 and LonP1 interactions per cell were counted in fiji software and reported as counts per cell.

### Statistical analysis

Student's *t*‐test was used for statistical analyses or one‐way ANOVA followed by the Bonferroni *post hoc* test. For immunofluorescence analysis, Pearson's correlation coefficient was used as a statistical test. The data in this study are presented as the mean, and error bars represent the SD. The data were acquired from at least three independent experiments. *P < 0.05 was considered significant differences among the experimental groups. The software used was graphpad prism version 8 (GraphPad Software; La Jolla, CA, USA; www.graphpad.com).

## Results

### The NEK5 mitochondrial protein‐interacting network

In our previous studies, we demonstrated that NEK5 interacts with mitochondrial proteins COX11 and MTX2, and its depletion leads to altered oxygen consumption rates [8]. To further explore the NEK5 mitochondrial interaction network, NEK5 was IP from crude mitochondria of 293T Flp‐In‐T‐Rex cells stably expressing wild‐type NEK5 and the results were analyzed by mass spectrometry (IP‐LC‐MS/MS) [33]. The analysis of NEK5 mitochondrial network (Table S1) showed proteins directly involved in metabolism, such as GPD1L, IRS4, GAPDH, AK2, TPI1, CTSD, and OXPHOS proteins such as SDHB (Fig. S1A). Pathway‐enriched analysis [34] of NEK5 mitochondrial proteome showed that signaling pathways such macroautophagy, glucose metabolism, negative cell‐cycle regulation, and regulation of RNA splicing are augmented in NEK5 mitochondrial network (Fig. S1B). Moreover, two mitochondrial matrix proteases that are involved in mtDNA remodeling, CLPP and LonP1, were also co‐IP along NEK5.

Some of NEK family members have already been described to participate in nuclear DDR [1, 2] and in mitochondrial function [8, 10, 12, 17]. Since NEK5 has been demonstrated to be involved in DDR through the interaction with topoisomerase IIβ, a mitochondrial topoisomerase isoform [4], and, as mentioned, has previously been related to mitochondrial functions, those preliminary results led us to investigate further the possible roles of NEK5 in mtDNA and its interaction with LonP1(Fig. 1B).

### NEK5 interacts with LonP1 and regulates its expression

LonP1 is a mitochondrial ATP‐dependent serine protease involved in mitochondrial functions and maintenance [35]. One of the main LonP1 functions is related to mtDNA replication and transcription, due to, especially, its selective degradation of TFAM [24]. LonP1 also specifically degrades other regulatory proteins such as mitochondrial steroidogenic acute regulatory (StAR) protein, helicase Twinkle (TWNK), and the DNA polymerase‐γ [36]. Studies revealed that LonP1 also plays a critical function in tumor cells by controlling bioenergetics and its upregulation induces profound changes in mitochondrial protein complexes, leading to the inactivation of mitochondrial respiration and favoring the glycolytic switch [37].

**Fig. 2:**
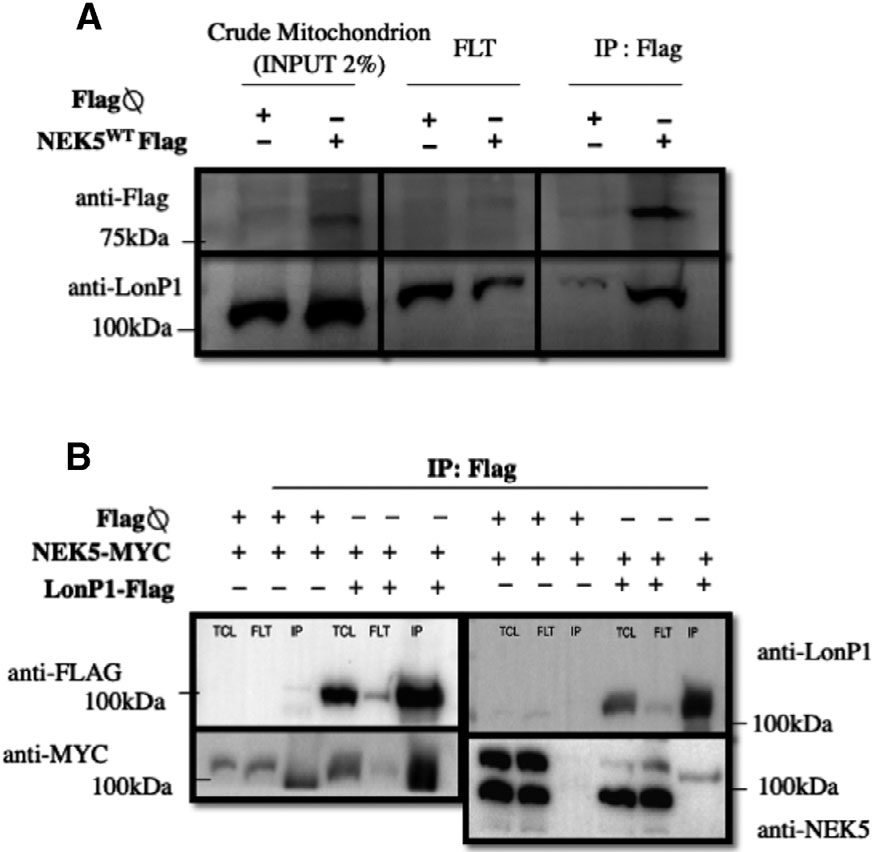
NEK5 interacts with LONP1 in the mitochondria and regulates its expression. (A) Endogenous LonP1 was co‐IP along with NEK5 from crude mitochondrial fraction from NEK5^WT^ cells using Flag as a bait. INPUT: crude mitochondrion; FLT: flow through. (B**)** Reverse co‐IP showing LonP1‐NEK5 interaction: HEK293T cells were co‐transfected with pcDNA3.2‐LonP1‐flag and pBABE‐BioID‐NEK5^WT^ MYC (~ 125 kDa) pcDNA3.2flag ⍉ or pBABE‐BioID‐NEK5^WT^ MYC (control); IP was performed using flag tag as a bait. TCL: total cell lysate; FLT: flow through.

**Fig. 3:**
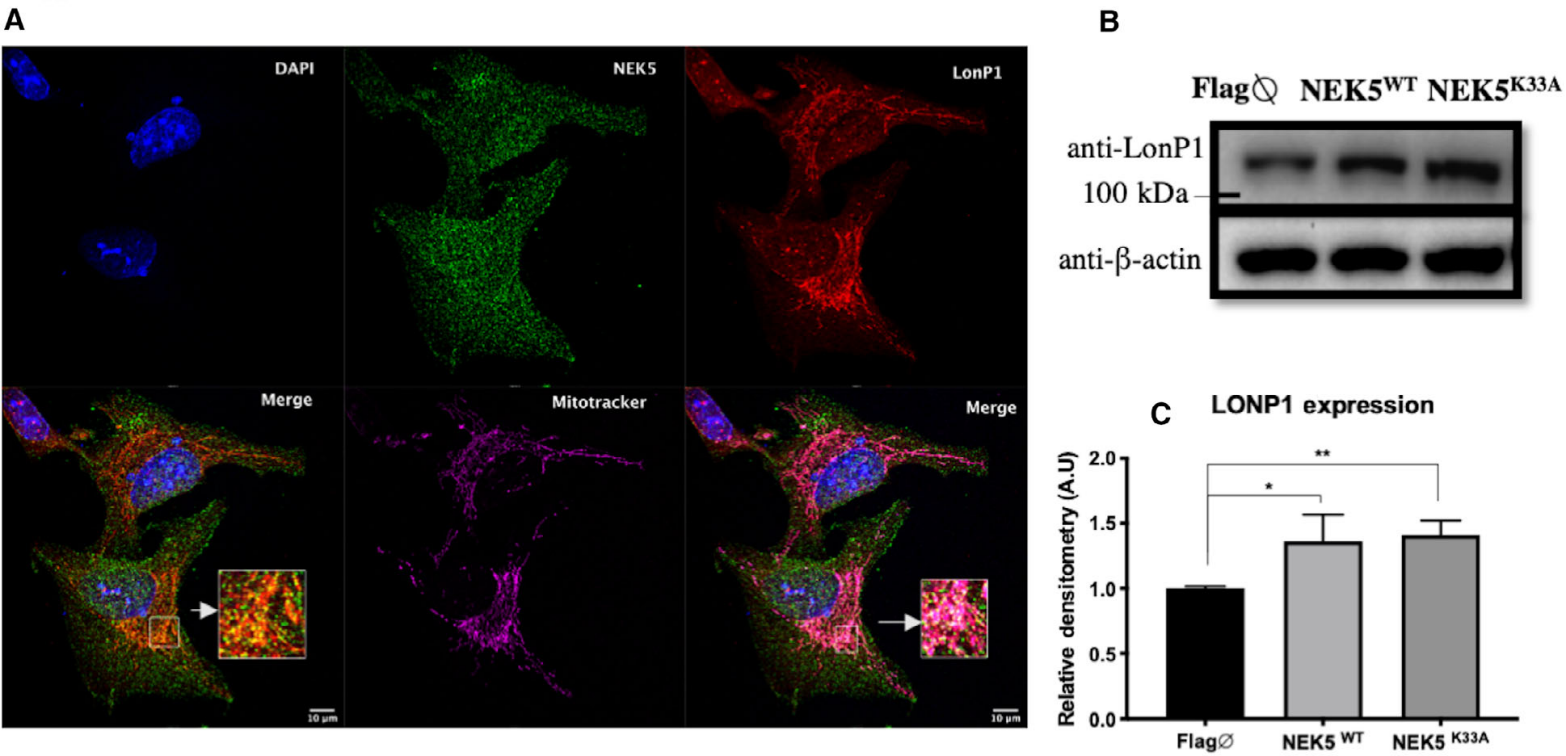
NEK5 co‐localizes with LonP1 in the mitochondria and regulates its expression. (A) Immunofluorescence analysis showing the co‐localization of NEK5 and LonP1, and the co‐localization of both proteins within the mitochondria, evidenced by co‐localization of NEK5 and LonP1 with MitoTracker™. Scale bar—10 µm. (B and C) LonP1 protein expression levels in response to NEK5^WT^ and NEK5^K33A^ overexpression, both wild‐type and kinase‐dead NEK5 lead to a significant LonP1 upregulation at protein level. The average of three replicates is represented, and the bar indicates SD of *n* = 3. One‐way ANOVA was used as statistical test. *P < 0.05, **P < 0.01.

We confirmed NEK5‐LonP1 interaction by Co‐IP in crude mitochondrial extract (Fig. S2A,B) from NEK5^WT^ (NEK5 wild‐type) Flp‐In‐expressing cells, using Flag‐NEK5 as a bait (Fig. 2A). We also demonstrated LonP1‐NEK5 interaction by reverse co‐IP using LonP1‐Flag as a bait, in HEK293T cells (Fig. 2B). LonP1 also interacts with NEK5^K33A^ (*kinase‐dead* NEK5), demonstrated by co‐IP in a total cell lysate (Fig. S2C). Immunofluorescence analysis showed the co‐localization of NEK5 and LonP1 in mitochondria of U2OS cells (Fig. 3A), with a correlation coefficient of 0.75 (Pearson's *R*‐value). Taken together, those data suggest that NEK5 interacts with LonP1 and this interaction is likely to happen in the mitochondria.

We evaluated LonP1 protein level in response to NEK5 overexpression. An increase in LonP1 levels in cells expressing the catalytically active NEK5^WT^ and NEK5^K33A^ was observed (Fig. 3B,C). HEK293T cells expressing pcDNA3.2LonP1‐flag also show an increase in NEK5 protein expression, suggesting a feedback loop response of those two proteins (Fig. S3A,B).

**Fig. 4:**
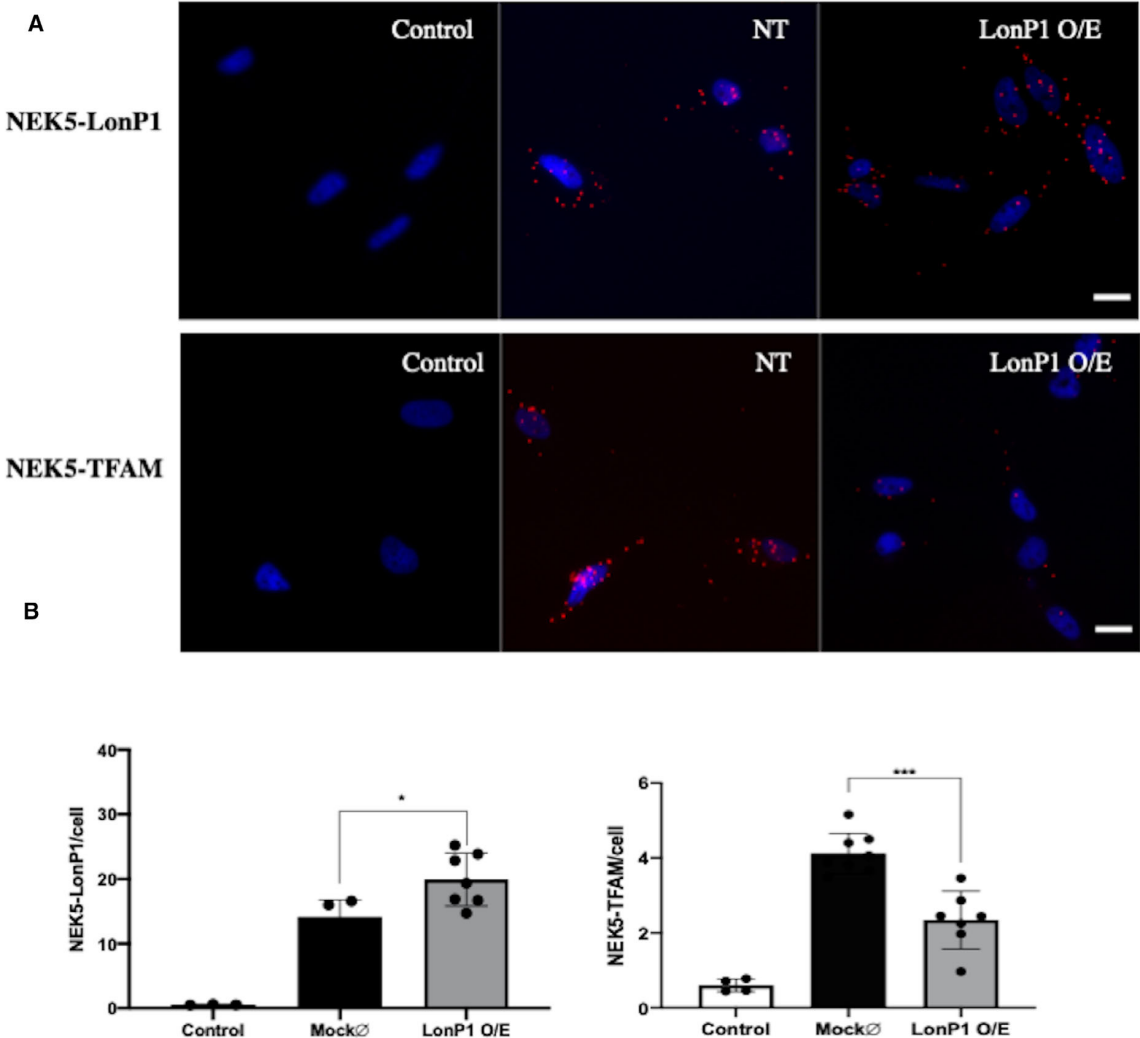
NEK5 participates in LonP1‐TFAM signaling axis. U2OS cells were transfected or not with pcDNA3.2LonP1‐flag vector and after 48 h were fixed with 3.7% PFA and submitted to PLA (A) PLA staining panel showing NEK5‐LonP1 interaction and NEK5‐TFAM interaction. NEK5 interacts with LonP1 endogenously, and upon LonP1 overexpression, this interaction is increased. NEK5 also interacts with TFAM endogenously, and upon LonP1 overexpression, this interaction is decreased, as LonP1 selective degrades TFAM. (B) Representative plots of NEK5‐LonP1 (left) and NEK5‐TFAM (right) interaction dot quantification. Scale bar—25 µm. The average of three replicates is represented, and the bar indicates SD of *n* = 3. One‐way ANOVA was used as statistical test. *P < 0.05, ***P < 0.001.

It is well established that LonP1 participates directly in mtDNA maintenance due to its ability to bind at specific regions of the mitochondrial nucleoids and also by the degradation of specific mtDNA core proteins, therefore controlling and regulating mtDNA replication, translation, and allowing access to mtDNA by specific degradation of mitochondrial nucleoid‐binding proteins [24, 38, 39, 40]. In order to test whether NEK5 can participate in the LonP1‐TFAM interaction axis, we employed PLA in U2OS cells. NEK5 is physically close to TFAM and LonP1, evidenced by positive PLA dots, pointing out to an interaction of these proteins. We next overexpressed pcDNA3.2 LonP1‐flag in U2OS cells and evaluate the dynamic of those interactions using PLA assay. Overexpression of LonP1 is expected to decrease TFAM levels, as LonP1 specifically degrades TFAM [24]. Upon LonP1 overexpression, NEK5‐LonP1 interaction increased when compared to nontransfected cells, and NEK5‐TFAM interaction decreased (probably due to the degradation of TFAM by LonP1), according to what we hypothesized [41] (Fig. 4A,B). Taken together, the data suggest that NEK5 interacts with TFAM and LonP1 and might participate in this signaling axis. The mechanisms underlying these interactions are still under investigation.

### NEK5^WT^ and NEK5^K33A^ overexpression led to altered mtDNA integrity

TFAM, one of the core components of mtDNA nucleoid, can bind to normal and damaged DNA, limiting access to base excision repair (BER) proteins, and that BER activity in mitochondria is affected by the modulation of the TFAM:mtDNA affinity by interacting proteins [42], being LonP1 the main regulator of TFAM:mtDNA levels [24]. As many mitotic kinases might have a similar role in nuclear and mtDNA [43, 44, 45] and NEK5 has already been linked with nuclear DDR, we thus hypothesized whether NEK5‐LonP1 interaction may affect mtDNA integrity and copy number.

**Fig. 5:**
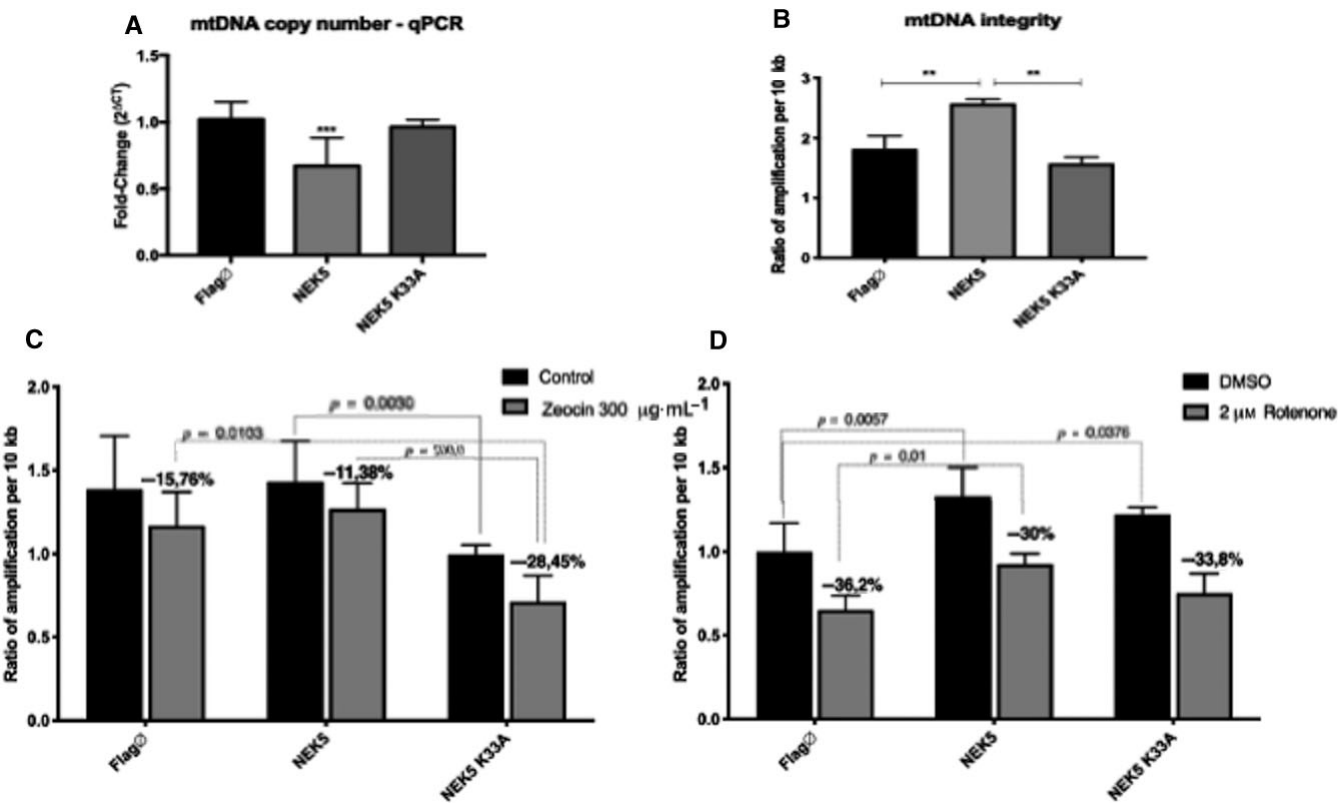
NEK5 kinase activity is required for mtDNA integrity maintenance. (A) mtDNA copy number evaluation by qPCR. The analysis showed the NEK5^WT^ cells tend to have less copies of mtDNA. (B) Evaluation of mtDNA integrity using LX‐PCR. NEK5^WT^ cells showed an increase in mtDNA integrity compared with both control and NEK5^K33A^. (C) mtDNA integrity evaluation after zeocin and (D) rotenone treatment. NEK5^WT^ demonstrated to be more resistant to both zeocin and rotenone treatment when compared to control and NEK5^K33A^ cells. The average of three replicates is represented, and the bar indicates SD of *n* = 3. Student's *t*‐test followed by Bonferroni's *post hoc* was used as statistical test. **P < 0.01, ***P < 0.001.

To evaluate the overall integrity of the mtDNA, we used long‐extension PCR (LX‐PCR), while qPCR was used to access mtDNA copy number in NEK5^WT^ and NEK5^K33A^ expressing cells. NEK5^WT^ cells showed an increase in overall mtDNA integrity and a small decrease in mtDNA copy number (Fig. 5A,B). In contrast, NEK5^K33A^ showed a decreased mtDNA integrity and no differences in mtDNA copy number when compared to NEK5^WT^ cells, suggesting that NEK5 kinase activity may improve mtDNA overall integrity. These results raise the possibility of NEK5 kinase participation in mtDNA maintenance and replication.

Next, we challenged NEK5 cells with zeocin (bleomycin analog), which causes nuclear and mtDNA damage [46], and rotenone, which leads to oxidative damage in mtDNA [47], and re‐evaluated mtDNA integrity. NEK5^K33A^ cells showed not only diminished mtDNA integrity in untreated cells but also more damage in mtDNA when exposed to zeocin. While control (Flag ∅) and NEK5^WT^ expressing cells showed 15.76 ± 0.317% and 11.38 ± 0.240% decrease in mtDNA integrity, respectively, upon treatment, NEK5^K33A^ presented 28.45 ± 0.058% decrease in mtDNA integrity (~ 40% more damage when compared to NEK5^WT^ cells), which reinforces our hypothesis of NEK5 kinase activity dependency in mtDNA maintenance (Fig. 5C). We observed the same phenotype upon oxidative mtDNA damage, where NEK5^WT^ cells seem to be more resistant to rotenone treatment compared to control (Flag ∅) and to NEK5^K33A^ expressing cells (Fig. 5D).

**Fig. 6:**
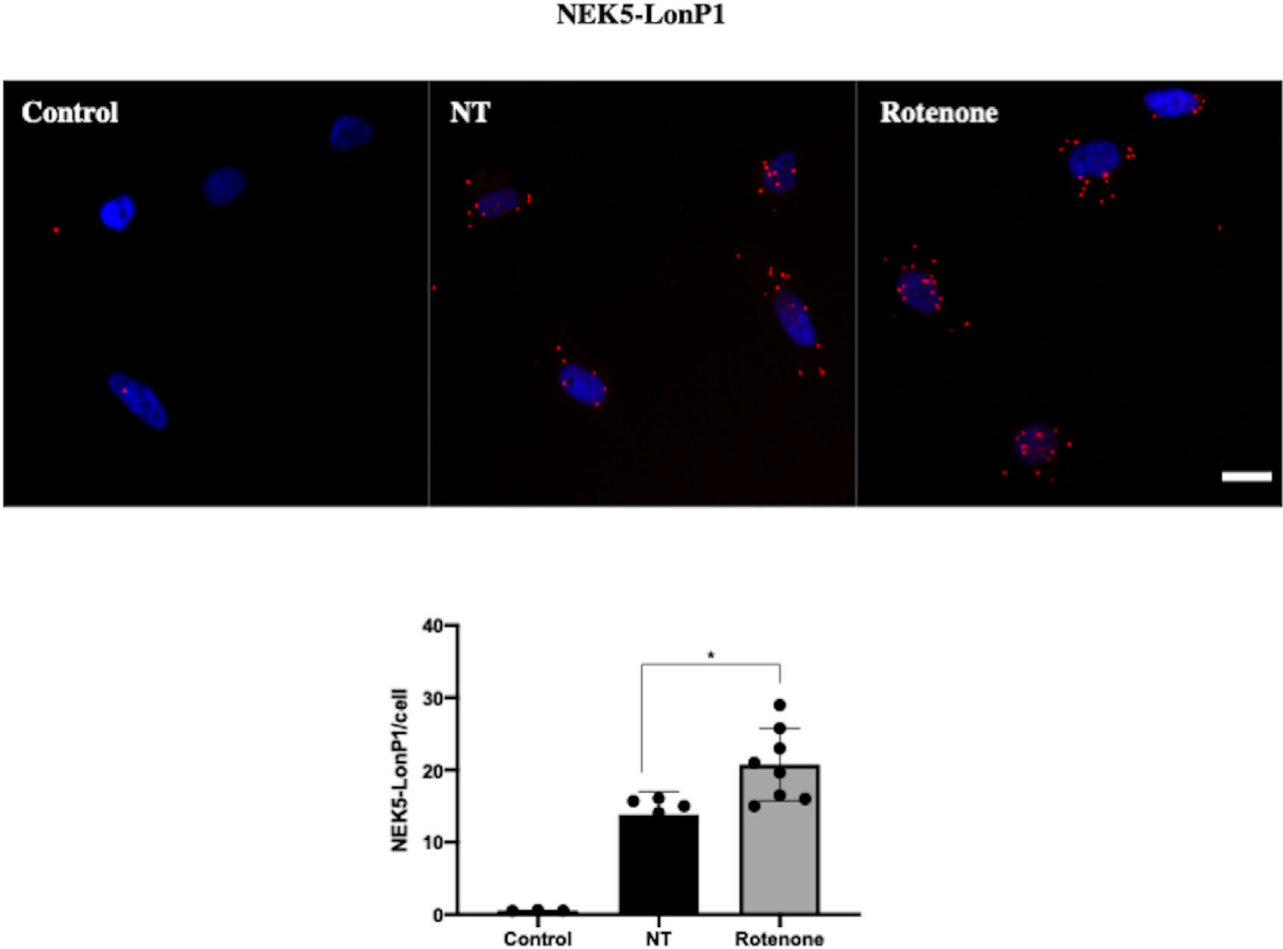
NEK5‐LonP1 interaction increases upon rotenone treatment. Upon oxidative damage, NEK5‐LonP1 increases, suggesting that the effect of NEK5 in mtDNA integrity might be related to this interaction. U2OS cells were treated or not with rotenone 2 μm for 1 h, fixed with 3.7% PFA and then submitted to PLA. Figures are representative of at least three different acquired images. Scale bar—25 µm. The average of three replicates is represented, and the bar indicates SD of *n* = 3. One‐way ANOVA was used as statistical test. *P < 0.05.

Next, we evaluate NEK5‐LonP1 interaction upon rotenone treatment using PLA in U2OS cells. We observed an increase in PLA dots upon treatment with rotenone, implying an increase in NEK5‐LonP1 interaction when cells are subjected to oxidative damage (Fig. 6). Those results suggest that NEK5 phenotype upon mtDNA integrity might be related to its interaction with LonP1.

In order to test whether the maintenance of mtDNA integrity in NEK5 cells is related to resistance to mitochondrial oxidants (mtROS)—the main source of mtDNA damage—we evaluate the amount of mtROS upon antimycin A treatment by flow cytometry using MitoSOX™ probe. The analysis showed that NEK5^WT^ cells tend to be more resistant to antimycin A treatment although not statistically significant (Fig. S4). These results suggest that the improvement in mtDNA integrity is not likely to be related exclusively to mtROS production and are in agreement with Hanchuk *et al*. (2015), which observed a reduction in total cellular ROS under basal conditions in cells overexpressing NEK5 and also resistance to ROS increase induced by H_2_O_2_ or thapsigargin [8].

### NEK5 alters the expression of genes related to mtBER

**Fig. 7:**
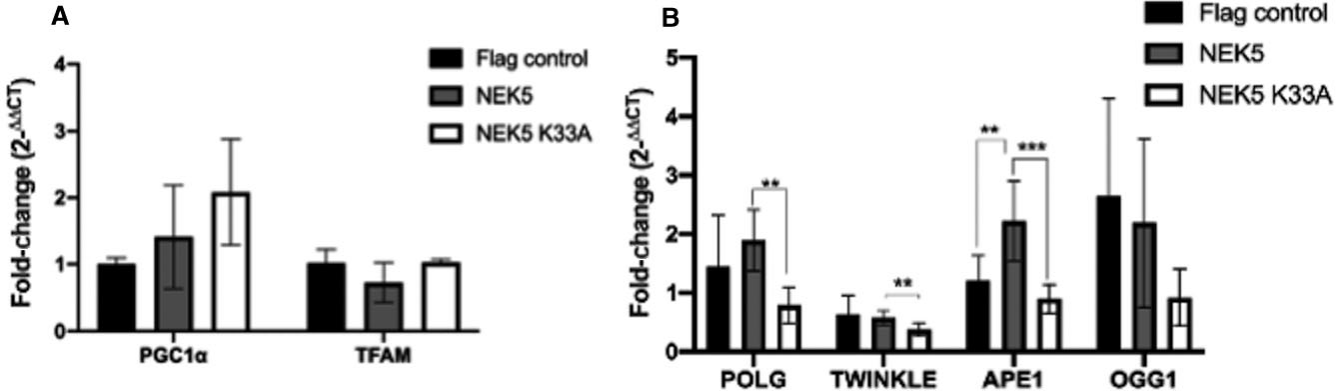
NEK5 affects the expression of mtDNA BER genes but not genes related to biogenesis. (A) The qPCR analysis of genes related to mitochondrial biogenesis demonstrated that NEK5 does not significantly alter the expression of TFAM or PGC1a; (B) NEK5 regulates mtBER and mtDNA replication genes in a kinase‐dependent manner: POLG, TWINKLE, and APE1 gene levels are increased in NEK5^WT^ cells compared with NEK5^K33A^; APE1 is also overexpressed in NEK5^WT^ when compared to control cells. The average of three replicates is represented, and the bar indicates SD of *n* = 3. Student's *t*‐test followed by Bonferroni's *post hoc* was used as statistical test. **P < 0.01, ***P < 0.001.

We hypothesized that the increase in mtDNA integrity in NEK5 cells could be related to mtDNA turnover and mitochondrial biogenesis, as NEK5^WT^ cells also showed a slight decrease in mtDNA copy number; thus, the increase in integrity would be a consequence of mtDNA turnover. TFAM is highly related to mtDNA content, degradation, and, consequently, turnover [48]; PGC‐1α is a major regulator of mitochondrial biogenesis and activates different transcription factors, including nuclear respiratory factor 1 and nuclear respiratory factor 2, which also activates TFAM [49, 50]. We evaluate the expression level of those genes and both, PGC1α and TFAM, do not vary significantly although PGC1α tend to be more expressed in NEK5^WT^ and NEK5^K33A^ cells (Fig. 7A); thus, NEK5 may not directly affect mtDNA turnover, and the increase in mtDNA integrity in NEK5^WT^ cells is likely to be related to an overall improvement in mtDNA maintenance.

The short‐patch BER was the first described DNA repair pathway in mammalian mitochondria, but nowadays many other DNA repair pathways such as nucleotide excision repair, mismatch repair, and long‐patch BER have already been described to take place also in mtDNA [51]. Thus, to further test the hypothesis that NEK5 participates in mtDNA maintenance we analyzed mRNA levels of genes related to mtDNA BER and replication by qPCR, such as POLγ, TWINKLE, OGG1, and APE1 in NEK5 cells. The AP endonuclease 1 (APE1) exists both in the nucleus and in mitochondria and is responsible for the removal of AP sites throughout the BER pathways. APE1 can regulate different transcription factors and inhibit ROS production as a way of controlling redox status in the cells and is also subject to different post‐translational modifications and phosphorylation in repair activity [52, 53]. POLγ is the only DNA polymerase in mitochondria, which assumes sole responsibility for DNA synthesis in all replication, recombination, and repair transactions and, as APE1, is encoded in the nucleus [53, 54]. Both POLγ and APE1 are downregulated in NEK5^K33A^ cells, and NEK5^WT^ cells showed an upregulation in APE1 (Fig. 7B). This could be related to a potential NEK5 activity in BER regulation, once both proteins are crucial in mitochondrial base excision repair (mtBER). The bifunctional DNA glycosylase OGG1 catalyzes the first steps in BER of oxidized purines, while TWINKLE is the main replicative helicase in mtDNA replication [42, 55]. Here, we observed a decreased expression of TWINKLE mRNA in NEK5^K33A^ cells but no differences in NEK5^WT^; OGG1 expression showed no significant differences. Taken together, these results open new insights about NEK5 biological roles, suggesting a possible involvement of NEK5, likely related to its kinase activity in mitochondrial DDR.

### NEK5 expression alters mitochondrial essential functions

Approximately 99% of mitochondrial proteins are encoded by the nuclear genome, being only a small set of 37 genes encoded by the mitochondrial genome in humans [56]. Throughout evolution, most mitochondrial genetic information had been transferred to the nucleus; however, mitochondria have retained its vestigial genome, allowing a form of genomic symbiosis through which mitochondria maintain a degree of cellular control, communicating with the nucleus through an incompletely understood series of retrograde signal [57].

In addition to this genomic nuclear–mitochondrial communication, mitochondria are highly dynamic organelles and this characteristic is related to its homeostasis, function, or dysfunction. This dynamic includes fusion and fission, biogenesis, mitophagy, mitochondrial mass, and activity and determines the balance between mitochondrial energy production and cell death programs [58]. Hence, there is a relationship between the translation of the mitochondrial genes, and coordination of nuclear‐encoded genes and mitochondrial functions.

**Fig. 8:**
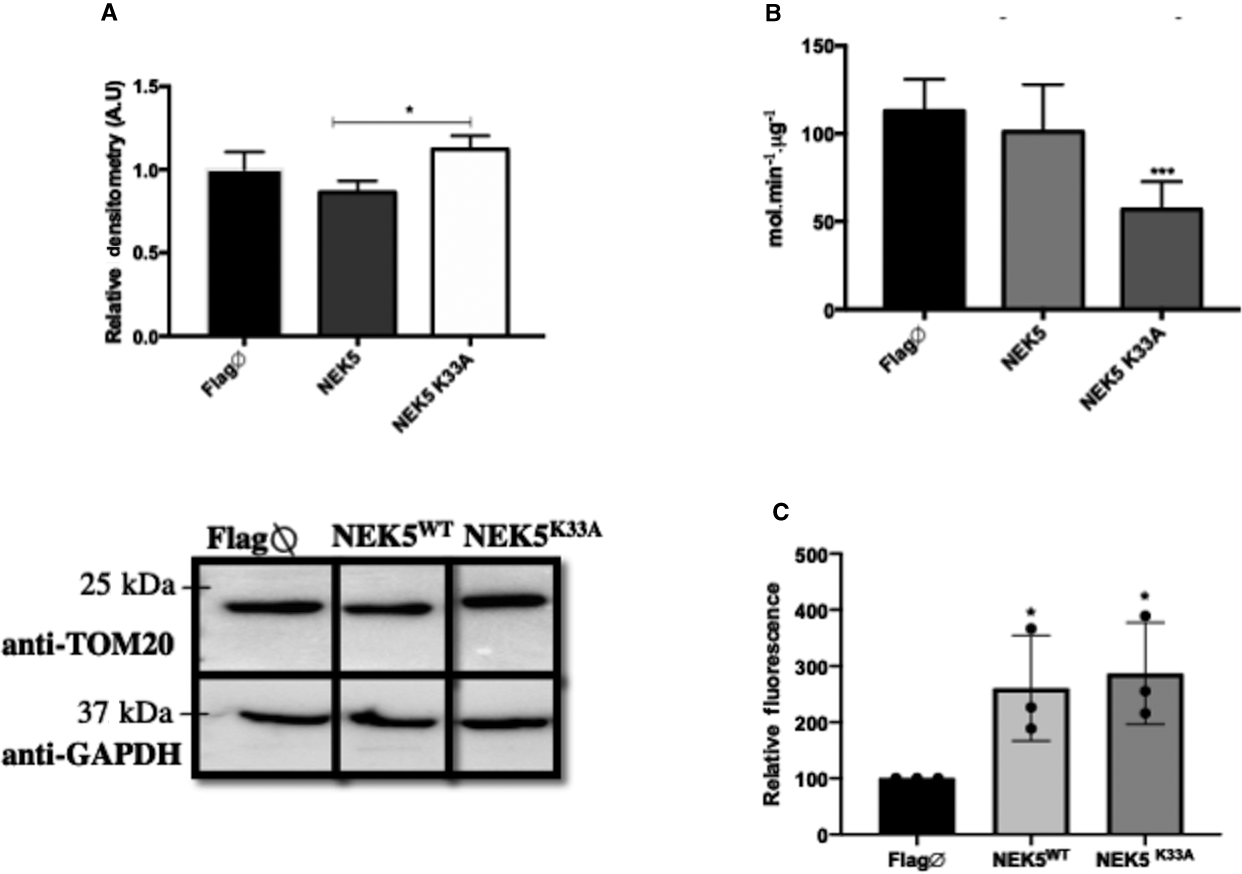
Mitochondrial mass and functionality are altered in NEK5^WT^ and NEK5^K33A^ cells. (A) Analysis of TOM20 protein expression, mitochondrial mass marker in NEK5 cells, and (B) measurement of citrase synthase activity. NEK5^WT^ showed a slightly decrease in TOM20 expression but no significant difference in CS activity. NEK5^K33A^ showed a significant decrease in CS activity and no differences in TOM20 expression, which suggests a deficiency in mitochondrial functionality due to the lack of NEK5 kinase activity. (C) flow cytometry analysis of ∆ψmt using TMRE™ probe showed an increase in ∆ψmt in both NEK5^WT^ and NEK5^K33A^. The average of three replicates is represented, and the bar indicates SD of *n* = 3. One‐way ANOVA was used as statistical test. *P < 0.05, ***P < 0.001.

To evaluate whether NEK5 expression alters other mitochondrial functions that might be linked to the observed increase in mtDNA integrity, we measure some mitochondrial key features such as total mitochondrial mass, CS activity, and ΔΨm. In NEK5^WT^ cells, we observed a decrease in mitochondrial mass (Fig. 8A) evidenced by the decrease in TOM20 expression, although the CS activity, a valuable marker of mitochondrial content and functionality [59], is not altered; a decrease in CS activity was observed in NEK5^K33A^ cells (Fig. 8B). Next, we evaluate ∆ψmt using TMRE™ probe and CCCP (decoupling agent) as a positive control. There are three fundamental functions driven by the ∆ψmt: ATP generation, Ca^2+^ uptake and storage, and the generation and detoxification of ROS [60], being a valuable information to access mitochondrial energy production. Both NEK5^WT^ and NEK5^K33A^ cells showed an increase in ∆ψmt compared to control (Fig. 8C). Interestingly, NEK5 ^WT^ had shown a decrease in complex II and complex IV respiration rate, but no significant change in basal respiration [8], and thus, the increase in ∆ψmt might be related to a compensation due to decreased activity on those complexes and also due to the decrease in mitochondrial mass in NEK5^WT^.

## Discussion

In our previous study, we demonstrated that NEK5 interacts with two mitochondrial proteins and both depletion and expression alter mitochondrial respiration. We then decided to further investigate the possible roles of NEK5 in mitochondrial homeostasis by exploring its mitochondrial interaction network. Among the putative partners of NEK5 in mitochondria, LonP1 was particularly interesting due to the number of hits in mass spectrometry analysis and the functions associated with this protease. Likewise, pathway‐enriched analysis of NEK5 mitochondrial proteome showed the potential involvement of NEK5 signaling in the regulation of RNA splicing in mitochondria, an event also regulated by LonP1. The mitochondrial LonP1 protease has been identified as an integral nucleoid core factor in human mitochondria, and it is involved in processes such as selective protein turnover (including ribosomal proteins), ATP‐dependent degradation of misfolded or damaged mitochondrial proteins, chaperone‐like function, assembly of mitochondrial complexes, association with mitochondrial nucleoids, and specific interaction with RNA [38]. LonP1 has the ability to bind mtDNA, specifically in sites where replication and transcription of mtDNA are initiated; this binding needs conformational changes in LonP1 itself, which are negatively regulated by phosphorylation [61]. The regulation of TFAM levels by LonP1 protease, as already mentioned in this study, is another well‐established role for LonP1. In cells with normal mtDNA levels, TFAM is phosphorylated within its HMG box 1 (HMG1) by cAMP‐dependent protein kinase (PKA) in mitochondria and this phosphorylation impairs TFAM binding to DNA to drive transcription activation [24]. TFAM is released from DNA upon phosphorylation by PKA, and DNA‐free TFAM, whether phosphorylated or not, is a LonP1 protease substrate. By controlling the levels of unassociated TFAM, LonP1 is one of the agents responsible for controlling mitochondrial nucleoid dynamics [62]. Here, we demonstrated that NEK5^WT^ and NEK5^K33A^ interact with LonP1, more likely within the mitochondria, and that both NEK5^WT^ and NEK5^K33A^ overexpression increased LonP1 protein levels. We also evaluated the possibility of NEK5 participation in the LonP1‐TFAM interaction axis. Using PLA, we demonstrated that NEK5 endogenously interacts with both LonP1 and TFAM, and upon LonP1 overexpression, NEK5‐LonP1 interaction increases (positive PLA dots) while NEK5‐TFAM interaction decreases, likely due to TFAM degradation by LonP1. The potential signaling module encompassing NEK5‐LonP1‐TFAM is under investigation.

Considering NEK5‐LonP1‐TFAM axis interaction and proximity (therefore mitochondrial nucleoids proximity), and NEK5 involvement with nuclear DDR, we decided to investigate the potential role of NEK5 in mtDNA maintenance and mitochondrial DDR. We observed that the expression of the NEK5^K33A^ mutant leads to a decrease in mtDNA integrity and might have an impaired mtDNA damage response when compared to NEK5^WT^. Not only cells expressing NEK5^WT^ showed a greater mtDNA integrity but also demonstrated to be more resistant to zeocin and rotenone treatment when compared both to control and NEK5^K33A^. We tested whether NEK5‐LonP1 interaction could be affected by oxidative damage in mtDNA, caused by rotenone, using PLA in U2OS cells. We observed that, upon rotenone treatment, the interaction between NEK5‐LonP1 increases, suggesting that the possible role of NEK5 in mtDNA maintenance and repair could be related to its interaction with LonP1.

**Fig. 9:**
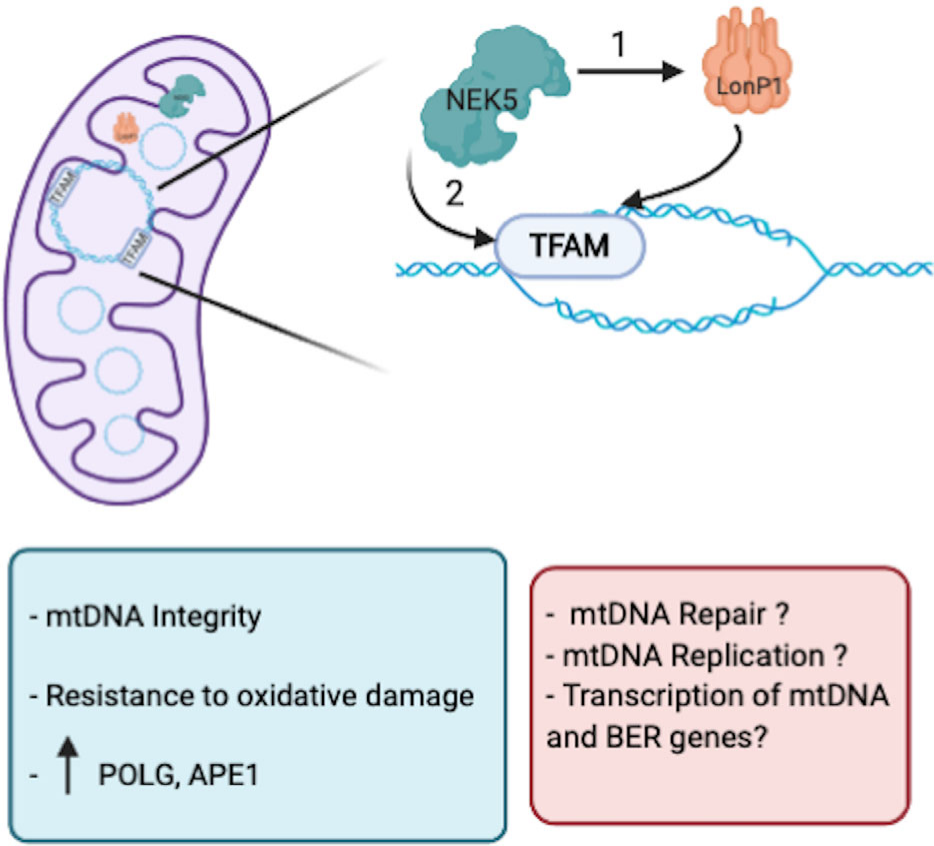
Proposed hypothesis underlying NEK5‐LonP1‐TFAM module. NEK5 interacts with both LonP1 and TFAM and might participate in the NEK5‐LonP1‐TFAM signaling axis by 1—activating LonP1 ATP‐dependent protease, leading to TFAM degradation, allowing mtDNA repair and replication or 2—interacting with TFAM (possible in a kinase‐dependent manner) leading to its specific degradation by LonP1, thus allowing mtDNA repair and replication under specific physiological circumstances. This interaction axis can be related to NEK5 roles in mtDNA, leading to increase in mtDNA integrity, resistance to oxidative damage, and upregulation of some of mtDNA BER genes. Blue box shows the results obtained in this work; orange box represents potential hypothesis regarding the results presented here.

As our observations showed that NEK5^WT^ is likely to be involved with mtDNA maintenance, we questioned whether the increase in mtDNA integrity in NEK5^WT^ could be related to a decrease in mitochondrial oxidants or even due to an improved and faster mtDNA turnover. For that, we evaluated mtROS, mtDNA content, and the expression of genes related to mitochondrial biogenesis, such as TFAM and PGC1α. Quantification of mtROS, mtDNA copy number, and TFAM and PGC1α gene levels showed no statistically significant differences in NEK5^WT^ compared with control, demonstrating that the increase in mtDNA integrity could be related to a mtDNA damage response exclusively. Therefore, here we demonstrated that NEK5^WT^ leads to an increase in mtDNA integrity, through still unknown mechanisms, and this phenotype is dependent on NEK5 kinase activity (see Fig. 9 for proposed hypothesis).

The analysis of mtDNA BER and replication genes suggests that NEK5 activity is required to regulate the expression of APE1, POLG, and TWINKLE helicase proteins, as these genes were upregulated upon NEK5^WT^ expression and significantly decreased in NEK5^K33A^ when compared to NEK5^WT^ wild‐type. OGG1 gene showed no significant difference upon neither NEK5^WT^ nor NEK5^K33A^ expression. The mitochondrial genome does not encode DNA repair or replication proteins, and thus, all mitochondrial BER proteins are splice variants, alternative translation–initiation products, or post‐translationally modified versions of the nuclear isoforms [54]. We still lack information on whether NEK5 regulation of those genes is a direct or an indirect consequence of NEK5 effect over mtDNA, or whether NEK5 participates in a nuclear–mitochondrial communication signaling pathway, although those results can be an interesting starting point for a deeper analysis of this possible regulation. The analysis of mitochondrial mass, functionality, and membrane potential in NEK5 cells suggested that NEK5 expression may also interfere in mitochondrial homeostasis. Upon NEK5^K33A^ expression, we observed a decrease in CS activity and no significant changes in mitochondrial mass marker, such as TOM20, suggesting that NEK5 kinase activity may be necessary in maintaining mitochondrial function; the increase in ΔΨm in NEK5^K33A^ in those cells might indicate a compensatory signaling in order to increase energy production [63], due to an increase in mitochondrial functionality (CS activity). Those altered mitochondrial functions in NEK5 cells could be consequences of its role in mtDNA or a direct regulation of mitochondrial metabolism, and those enquiries are already under investigation. Interestingly, some cell‐cycle kinases have already been described to relocate to mitochondria and regulate mitochondrial metabolism and bioenergetics. Cdk1, for example, relocates to mitochondria in irradiated cells, where it boosts ATP generation via phosphorylation of complex I, favoring DNA repair and cell survival [44]. Wang *et al*. (2014) demonstrated that a fraction of cell‐cycle cyclinB1/Cdk1 proteins localize into the mitochondrial matrix and phosphorylate a cluster of mitochondrial proteins, allowing the cells to sense and respond to an increased energy demand for G2/M transition, and subsequently to upregulate mitochondrial respiration for a successful cell‐cycle progression [64]. Aurora kinase A, which its role in mitotic progression has been extensively characterized, was shown to be located and imported into the mitochondrion in several human cancer cell lines, and to have an impact on mitochondrial dynamics and energy production [43]. Also, Mps1, a cell‐cycle kinase that regulates cell viability through its role in spindle assembly checkpoint, was found to be recruited to mitochondria by binding to voltage‐dependent anion channel 1 [45]. A substantial amount of energy, mostly produced by mitochondria, is required for cell‐cycle progression and DNA repair, but the mechanisms underlying the coordination of the mitochondrial respiration with cell‐cycle progression, especially the G2/M transition, and how the energy production contributes to DNA repair are not clearly established. So far, we still lack information if the occurrence of NEK family members in metabolic processes—such as mitochondrial functions—is related to the coordination of energy supply to cell cycle or maintenance of cell homeostasis, but indeed they seem to participate and regulate many metabolic pathways, and NEK5 could be one of these kinases.

## Conclusion

We demonstrated that NEK5 interacts with LonP1, more likely within the mitochondrial matrix, and that NEK5 overexpression, either *wild‐type* or *kinase dead*, leads to an increase in LonP1 expression. In addition, we showed that NEK5 might participate in a NEK5‐LonP1‐TFAM signaling module and the data suggest that NEK5 might be located at the mitochondrial nucleoids, due to its proximity/co‐localization with those proteins. We also demonstrated that NEK5 might be related to mtDNA maintenance and repair in a kinase‐dependent manner as NEK5^K33A^ mutant tended to be more affected by zeocin and rotenone treatment when compared to the wild‐type kinase. Moreover, this phenotype could be associated with NEK5‐LonP1 interaction since an increase in PLA signal between NEK5 and LonP1 was observed upon oxidative mtDNA damage. NEK5 might also modulate the expression of mitochondrial BER and replication genes, leading to the upregulation of POLG, TWINKLE, and APE1 genes in a kinase activity‐related manner.

Lastly, we showed that the lack of NEK5 kinase activity caused a decrease in mitochondrial function, evidenced by decreased mitochondrial CS activity and not mass in NEK5^K33A^ cells, reinforcing our hypothesis of a NEK5 role in mitochondrial function dependent on its kinase activity. Taken together, our data showed a new role for NEK5 kinase apart from cell cycle, and its involvement with mtDNA maintenance and mitochondrial essential functions.

## Conflict of interest

The authors declare no conflict of interest.

## Author contributions

TDMH performed the mass spectrometry experiment, and CCF performed all other experiments with the help of FLB, ALO, APO, and MPM. All former authors analyzed the data together with NCS‐P and JK. The project was conceived by JK and CCF, with contributions from NCS‐P and FLB. JK provided the grant support and supervised the overall project with the help of NCS‐P. CCF wrote the draft. All other authors contributed specific parts to the writing and improved the final version.

## Supporting information


**Table S1.** Table of significant proteins identified in IP LC‐MS/MS in NEK5 Cells. NEK5^WT^ was co‐IP from crude mitochondrion using flag‐tag antibody. Samples were submitted to LC‐MS/MS and were analyzed in Scaffold Q+ v.3.3.2. The table shows a summary the proteins identified in the mitochondrion fraction of flag‐NEK5^WT^ overexpressed cells with significant hits in MS analysis, Molecular Function and Biological Processes associated to those putative partners.

**Fig. S1.** NEK5 mitochondrial protein interacting network. A ‐ Interaction network of human NEK5 with potential mitochondrial partners identified by IP‐LC‐MS/MS. The proteomic data retrieved from IP‐LC‐MS/MS was submitted to the Integrated Interactome System (IIS) platform (National Laboratory of Biosciences, Campinas, Brazil) (Carazzolle *et al*., 2014). The protein network was assembled using Cytoscape 3.7.0 software (Shannon *et al*., 2003). B‐ Enriched pathway analysis of NEK5 mitochondrial interactome. The bioinformatic analysis shows that pathways such as Regulation of macroautophagy, Glucose Metabolism, negative cell‐cycle regulation and RNA –splicing regulation are up‐regulated in NEK5 mitochondrial network. The analysis was performed using Metascape (http://metascape.org).

**Fig. S2.** Confirmation of NEK5^WT^ and NEK5^K33A^ inducible expression and crude mitochondrion isolation. A ‐ Western Blotting showing NEK5 expression in Flp‐In™ T‐REx™ 293T Flag (Flag ⍉), Flp‐In™ T‐REx™ 293T Flag‐NEK5^WT^ (NEK5^WT^) and Flp‐In™ T‐REx™ 293T Flag‐NEK5^K33A^ (NEK5^K33A^). Cells were induced with 0, 1 and 2 μm of Tetracycline for 48 h and assayed for NEK5 expression using anti‐Flag antibody. B – Confirmation of crude mitochondrion isolation. PNS (Supernatant) contains both mitochondrion and nuclei fractions; CYTO (Cytosol) contains only the Cytosolic fraction; MITO (Mitochondria), contains crude mitochondrion fraction. TOM20 was used as mitochondrion marker and should be present at PNS and MITO fractions; GAPDH was used as a cytosolic marker and should be present in PNS and CYTO fractions only. C ‐ Endogenous LONP1 was co‐IP along with NEK5^K33A^ from total cell lysate from NEK5^K33A^ cells using Flag as a bait. TCL – Total Cell Lysate; FLT: Flow Through. The expression of NEK5^K33A^increases LonP1 protein levels, leading to the difference in the Total Cell Lysate loading observed in the IP.

**Fig. S3.** NEK5 expression level is increased upon LonP1 overexpression. A – NEK5 is upregulated in LonP1 overexpressed cells; relative Densitometry. B – Immunoblotting showing NEK5 protein level in HEK293T cells transfected with pcDNA3.2LonP1‐flag or pcDNA3.2flag ⍉. The average of three replicates is represented, and the bar indicates SD of *n* = 3. Student *T*‐test followed by Bonferroni post‐hoc was used as Statistical Test.

**Fig. S4.** Overexpression of NEK5 does not significantly affects mitochondrial oxidants production. Flow Cytometry analysis of mitochondrial oxidants utilizing MitoSOX™ probe. The results showed no significant changes in mtROS suggesting that the increase in mtDNA integrity in NEK5^WT^ are not related to mtROS. The average of three replicates is represented, and the bar indicates SD of *n* = 3. Student *T*‐test followed by Bonferroni post‐hoc was used as Statistical Test.

## Data Availability

The data that support the findings of this study are available in the body of the text and the Supporting information of this article. Complementary data that support the findings are also available from the corresponding author (jorgkoba@unicamp.br) upon reasonable request.
