# Supplementary material for: NEK5 interacts with LonP1 and its kinase activity is essential for the regulation of mitochondrial functions and mtDNA maintenance
Source: FEBS Open Bio. 2021 Feb 24;11(3):546–63. doi: 10.1002/2211-5463.13108 (PMC7931231; doi:10.1002/2211-5463.13108)
Supplement: Supplementary file 1 — Table S1. Table of significant proteins identified in IP LC‐MS/MS in NEK5 Cells. NEK5WT was co‐IP from crude mitochondrion using flag‐tag antibody. Samples were submitted to LC‐MS/MS and were analyzed in Scaffold Q+ v.3.3.2. The table shows a summary the proteins identified in the mitochondrion fraction of flag‐NEK5WT overexpressed cells with significant hits in MS analysis, Molecular Function and Biological Processes associated to those putative partners. [file FEB4-11-546-s001.docx]

**Supplementary files:**

**NEK5 interacts with LonP1 and its kinase activity is essential for the regulation of mitochondrial functions and mtDNA maintenance**

**By: Camila de Castro Ferezin, Fernanda Luisa Basei, Talita D. Melo-Hanchuk, Ana Luisa de Oliveira, Andressa Peres de Oliveira, Mateus P. Mori, Nadja C. de Souza-Pinto, Jörg Kobarg**

**Table S1:** **Table of proteins identified in IP LC-MS/MS in NEK5 Cells.** NEK5^WT^ was co-immuneprecipitated from crude mitochondrion using flag-tag antibody. Samples were submitted to LC-MS/MS and were analyzed in Scaffold Q + v.3.3.2. The table shows a summary the proteins identified in the mitochondrion fraction of flag-NEK5^WT^ overexpressed cells with significant hits in MS analysis, Molecular Function and Biological Processes associated to those putative partners.

| **Acess Number** | **Protein** | **Gene** | **Molecular Function** | **Biological Process** |
| --- | --- | --- | --- | --- |
| P21912 | Succinate dehydrogenase [ubiquinone] iron-sulfur subunit | SDHB | Oxidoreductase | Electron transport, Transport, Tricarboxylic acid cycle. |
| P04406 | Glyceraldehyde-3-phosphate dehydrogenase | GAPDH | Oxidoreductase, Transferase | Apoptosis, Glycolysis, Translation regulation |
| P54819-5 | Isoform 5 of Adenylate kinase 2 | AK2 | Kinase, Transferase | Interconversion of nucleotide di- and triphosphates. |
| P30041 | Peroxiredoxin-6 | PRDX6 | Cadherin binding, Calcium-independent phospholipase A2 activit, Glutathione peroxidase; Peroxidase activit, Peroxiredoxin activity, Phospholipase A2 activity, Protein homodimerization activity, Ubiquitin protein ligase binding | Cell redox homeostasis; Cellular oxidant detoxification; Cellular response to oxidative stress; Glycerophospholipid catabolic process; Hydrogen peroxide catabolic process; Positive regulation of mRNA splicing, via spliceosome; Response to oxidative stress |
| M0R208 | ATP-dependent Clp protease proteolytic subunit | CLPP | Serine-type endopeptidase activity | Hydrolase, Protease, Serine protease. |
| E9PDU6 | Calponin-3 | CNN3 | Actin binding, Calmodulin binding | Actomyosin structure organization |
| F5GZ27 (P36776) | Lon protease homolog | LONP1 | DNA-binding, Hydrolase, Protease, Serine protease | Aging, Cellular protein complex assembly, Cellular response to oxidative stress, Mitochondrial DNA metabolic process, Mitochondrial genome maintenance, Mitochondrion organization, Protein Quality Control, Proteolysis, Response to hypoxia. |
| Q14257 | Reticulocalbin-2 | RCN2 | Calcium ion binding | - |
| O14654 | Insulin receptor substrate 4 | IRS4 | Insulin receptor binding, Phosphatidylinositol 3-kinase binding, SH3/SH2 adaptor activity, Signal transducer activity | Insulin receptor signaling pathway, Regulation of lipid metabolic process, Signal transduction. |
| F8WD96 | Cathepsin D | CTSD | Aspartyl protease | aspartic-type endopeptidase activity |
| F5H897 | Heat shock protein 75 kDa, mitochondrial | TRAP1 | ATP binding; Protein kinase binding; RNA binding; Tumor necrosis factor receptor binding; Unfolded protein binding | Chaperone-mediated protein folding; Negative regulation of cellular respiration; Negative regulation of intrinsic apoptotic signaling pathway; Negative regulation of reactive oxygen species; Translational attenuation |
| O14618 | Copper chaperone for superoxide dismutase | CCS | Cadherin binding; Copper ion binding; Protein disulfide; Oxidoreductase activity; Superoxide dismutase activity; Superoxide dismutase; Copper chaperone activity; Zinc ion binding | Cellular response to oxidative stress; Metal ion transport;  Positive regulation of oxidoreductase activity;  Protein maturation by copper ion transfer; Removal of superoxide radicals; Central superoxide metabolic process |
| P53396 | ATP-citrate synthase | ACLY | ATP binding; ATP citrate synthase activity; Cofactor binding; Metal ion binding | Acetyl-CoA biosynthetic process; Cholesterol biosynthetic process; Citrate metabolic process; Coenzyme A metabolic process, Fatty acid biosynthetic process; Fatty-acyl-CoA biosynthetic process; Lipid biosynthetic process; Oxaloacetate metabolic process |
| Q8NBS9-2 | Isoform 2 of Thioredoxin domain-containing protein 5 | TXNDC5 | protein disulfide isomerase activity | Apoptotic cell clearance; Cell redox homeostasis; Negative regulation of apoptotic process, Neutrophil degranulation; Protein folding; Response to endoplasmic reticulum stress |
| P60174-4 | Triosephosphate isomerase | TPI1 | Isomerase | Gluconeogenesis, Glycolysis, Pentose shunt |
| P30041 | Peroxiredoxin-6 | PRDX6 | Antioxidant, Hydrolase, Oxidoreductase, Peroxidase | Lipid degradation; Lipid metabolism |
| F8W6E4 (Q8N335) | Glycerol-3-phosphate dehydrogenase | GPD1L | Oxidoreductase, sodium channel regulator activity | Carbohydrate metabolic process; Glycerol-3-phosphate catabolic process; NADH metabolic process; Negative regulation of peptidyl-serine phosphorylation; Negative regulation of protein kinase C signaling; Positive regulation of sodium ion transport; Regulation of heart rate; Regulation of sodium ion transmembrane transporter activity |


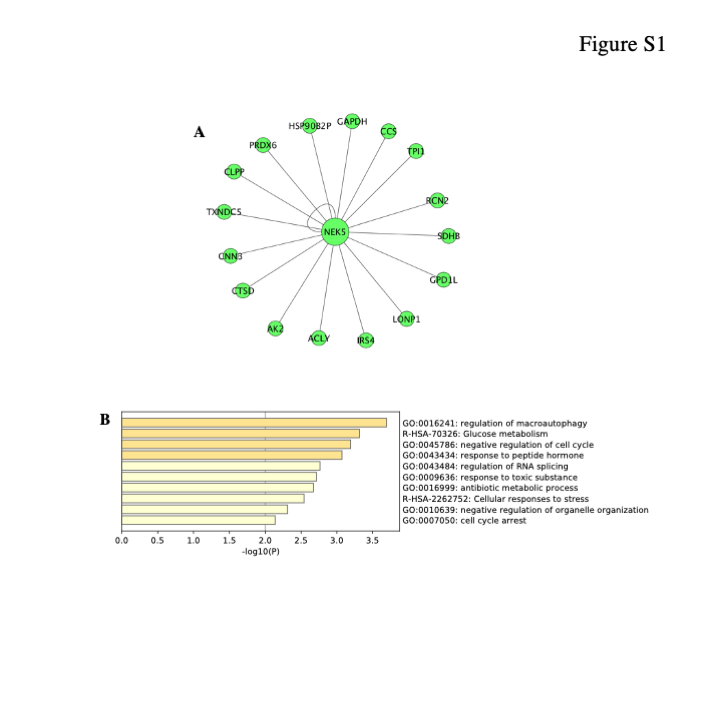


**Figure S1:** **NEK5 mitochondrial protein interacting network.** A - Interaction network of human NEK5 with potential mitochondrial partners identified by IP-LC-MS/MS. The proteomic data retrieved from IP-LC-MS/MS was submitted to the Integrated Interactome System (IIS) platform (National Laboratory of Biosciences, Campinas, Brazil) (Carazzolle et al., 2014). The protein network was assembled using Cytoscape 3.7.0 software (Shannon et al., 2003).

B- Enriched pathway analysis of NEK5 mitochondrial interactome. The bioinformatic analysis shows that pathways such as Regulation of macroautophay, Glucose Metabolism, negative cell-cycle regulation and RNA –splicing regulation are up-regulated in NEK5 mitochondrial network. The analysis was performed using Metascape (http://metascape.org).


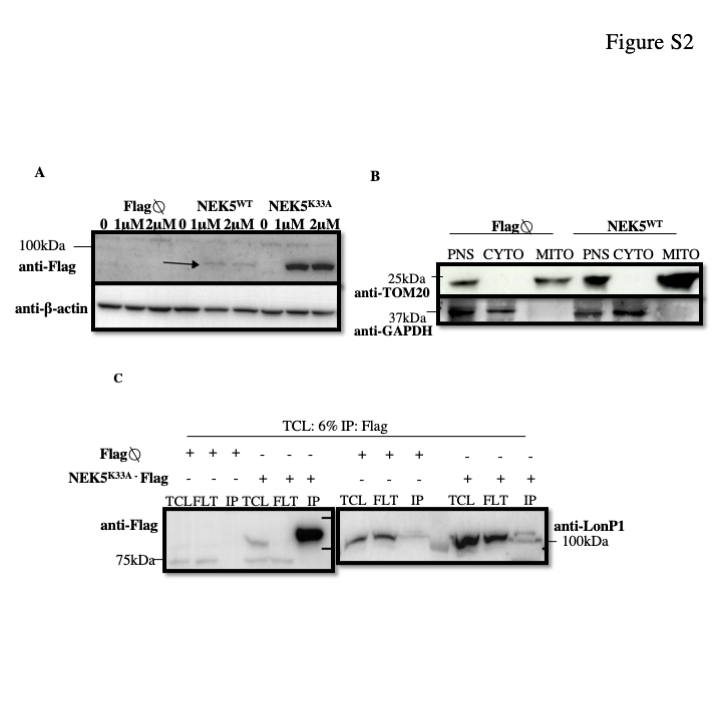


**Figure S2:** **Confirmation of NEK5^WT^ and NEK5^K33A^ inducible expression and crude mitochondrion isolation.** A - Western Blotting showing NEK5 expression in Flp-In™ T-REx™ 293T Flag (**Flag**$\boldsymbol{⍉}$***),*** Flp-In™ T-REx™ 293T Flag-NEK5^WT^ (NEK5^WT^) and Flp-In™ T-REx™ 293T Flag-NEK5^K33A^ (NEK5^K33A^). Cells were induced with 0, 1 and 2μM of Tetracycline for 48h and assayed for NEK5 expression using anti-Flag antibody. B – Confirmation of crude mitochondrion isolation. PNS (Supernatant) contains both mitochondrion and nuclei fractions; CYTO (Cytosol) contains only the Cytosolic fraction; MITO (Mitochondria), contains crude mitochondrion fraction. TOM20 was used as mitochondrion marker and should be present at PNS and MITO fractions; GAPDH was used as a cytosolic marker and should be present in PNS and CYTO fractions only. C - Endogenous LONP1 was co-immunoprecipitated along with NEK5^K33A^ from total cell lysate from NEK5^K33A^ cells using Flag as a bait. TCL – Total Cell Lysate; FLT: Flow Through; IP: Immunoprecipitated. The expression of NEK5^K33A^increases LonP1 protein levels, leading to the difference in the Total Cell Lysate loading observed in the IP.


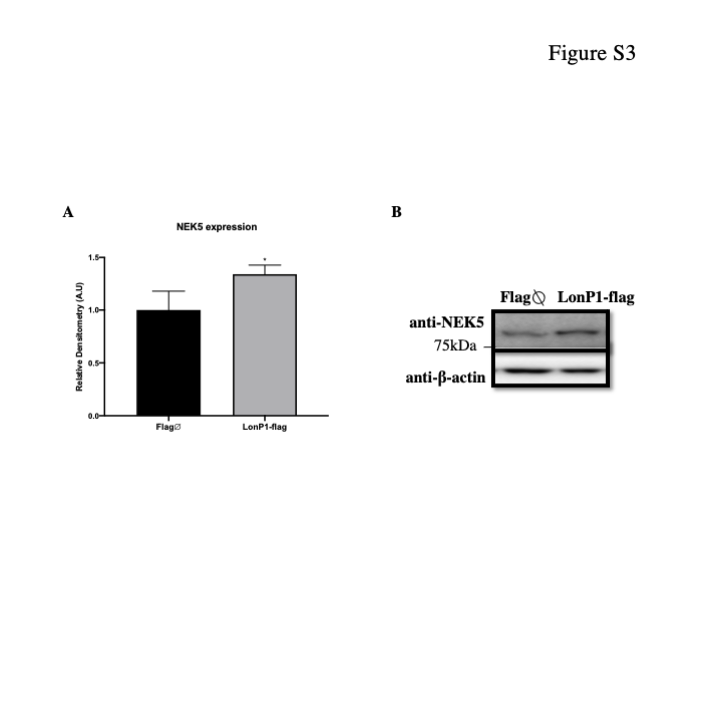


**Figure S3:** **NEK5 expression level is increased upon LonP1 overexpression.** A – NEK5 is upregulated in LonP1 overexpressed cells; relative Densitometry. B – Immunoblotting showing NEK5 protein level in HEK293T cells transfected with pcDNA3.2LonP1-flag or pcDNA3.2flag$\boldsymbol{⍉}$. The average of three replicates is represented, and the bar indicates SD of *n* = 3. Student T-test followed by Bonferroni post-hoc was used as Statistical Test.


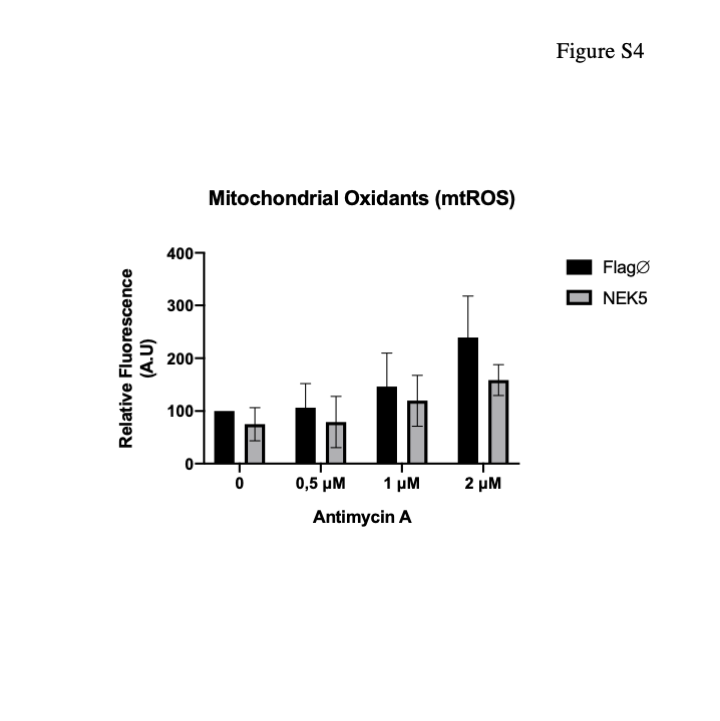


**Figure S4: Overexpression of NEK5 does not significantly affects mitochondrial oxidants production.** Flow Cytometry analysis of mitochondrial oxidants utilizing MitoSox™ probe. The results showed no significant changes in mtROS suggesting that the increase in mtDNA integrity in NEK5^WT^ are not related to mtROS. The average of three replicates is represented, and the bar indicates SD of *n* = 3. Student T-test followed by Bonferroni post-hoc was used as Statistical Test.
