# Supplementary figures and images for: NEK5 interacts with LonP1 and its kinase activity is essential for the regulation of mitochondrial functions and mtDNA maintenance
Source: FEBS Open Bio. 2021 Feb 24;11(3):546–63. doi: 10.1002/2211-5463.13108 (PMC7931231; doi:10.1002/2211-5463.13108)

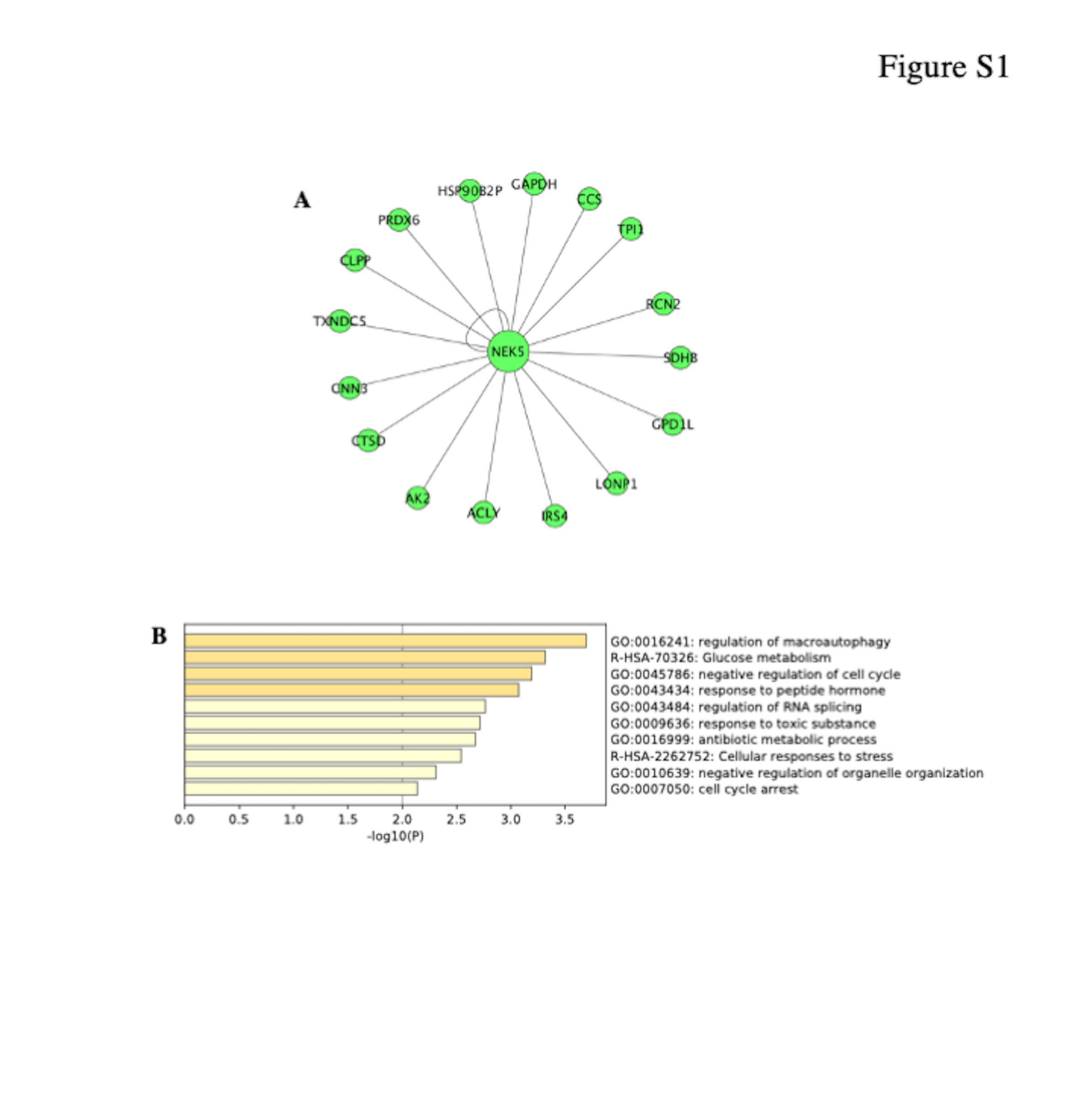

Supplement: Supplementary file 2 — Fig. S1. NEK5 mitochondrial protein interacting network. A ‐ Interaction network of human NEK5 with potential mitochondrial partners identified by IP‐LC‐MS/MS. The proteomic data retrieved from IP‐LC‐MS/MS was submitted to the Integrated Interactome System (IIS) platform (National Laboratory of Biosciences, Campinas, Brazil) (Carazzolle et al., 2014). The protein network was assembled using Cytoscape 3.7.0 software (Shannon et al., 2003). B‐ Enriched pathway analysis of NEK5 mitochondrial interactome. The bioinformatic analysis shows that pathways such as Regulation of macroautophagy, Glucose Metabolism, negative cell‐cycle regulation and RNA –splicing regulation are up‐regulated in NEK5 mitochondrial network. The analysis was performed using Metascape (http://metascape.org). [file FEB4-11-546-s002.tiff]

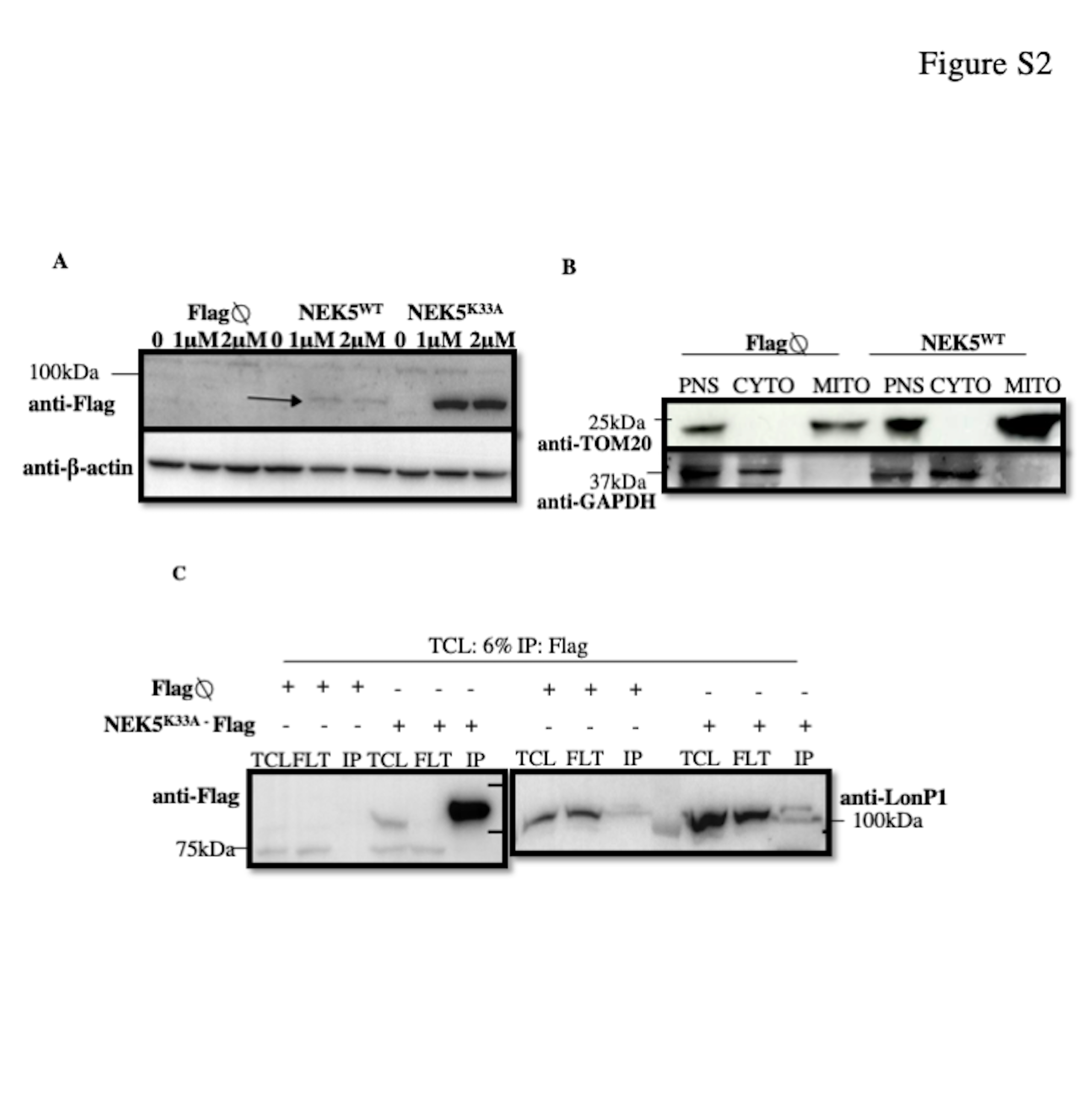

Supplement: Supplementary file 3 — Fig. S2. Confirmation of NEK5WT and NEK5K33A inducible expression and crude mitochondrion isolation. A ‐ Western Blotting showing NEK5 expression in Flp‐In™ T‐REx™ 293T Flag (Flag ⍉), Flp‐In™ T‐REx™ 293T Flag‐NEK5WT (NEK5WT) and Flp‐In™ T‐REx™ 293T Flag‐NEK5K33A (NEK5K33A). Cells were induced with 0, 1 and 2 μm of Tetracycline for 48 h and assayed for NEK5 expression using anti‐Flag antibody. B – Confirmation of crude mitochondrion isolation. PNS (Supernatant) contains both mitochondrion and nuclei fractions; CYTO (Cytosol) contains only the Cytosolic fraction; MITO (Mitochondria), contains crude mitochondrion fraction. TOM20 was used as mitochondrion marker and should be present at PNS and MITO fractions; GAPDH was used as a cytosolic marker and should be present in PNS and CYTO fractions only. C ‐ Endogenous LONP1 was co‐IP along with NEK5K33A from total cell lysate from NEK5K33A cells using Flag as a bait. TCL – Total Cell Lysate; FLT: Flow Through. The expression of NEK5K33Aincreases LonP1 protein levels, leading to the difference in the Total Cell Lysate loading observed in the IP. [file FEB4-11-546-s003.tiff]

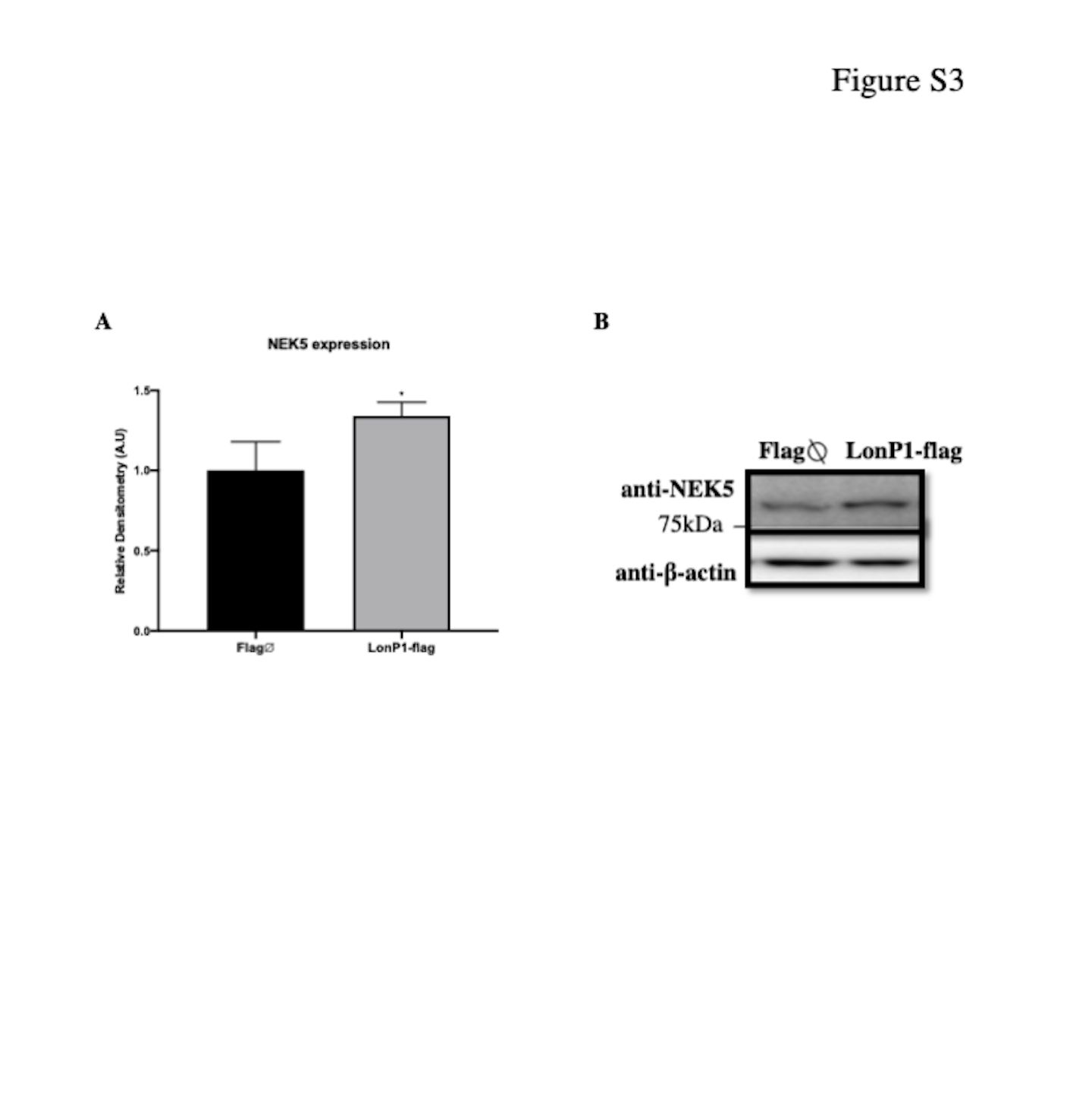

Supplement: Supplementary file 4 — Fig. S3. NEK5 expression level is increased upon LonP1 overexpression. A – NEK5 is upregulated in LonP1 overexpressed cells; relative Densitometry. B – Immunoblotting showing NEK5 protein level in HEK293T cells transfected with pcDNA3.2LonP1‐flag or pcDNA3.2flag ⍉. The average of three replicates is represented, and the bar indicates SD of n = 3. Student T‐test followed by Bonferroni post‐hoc was used as Statistical Test. [file FEB4-11-546-s004.tiff]

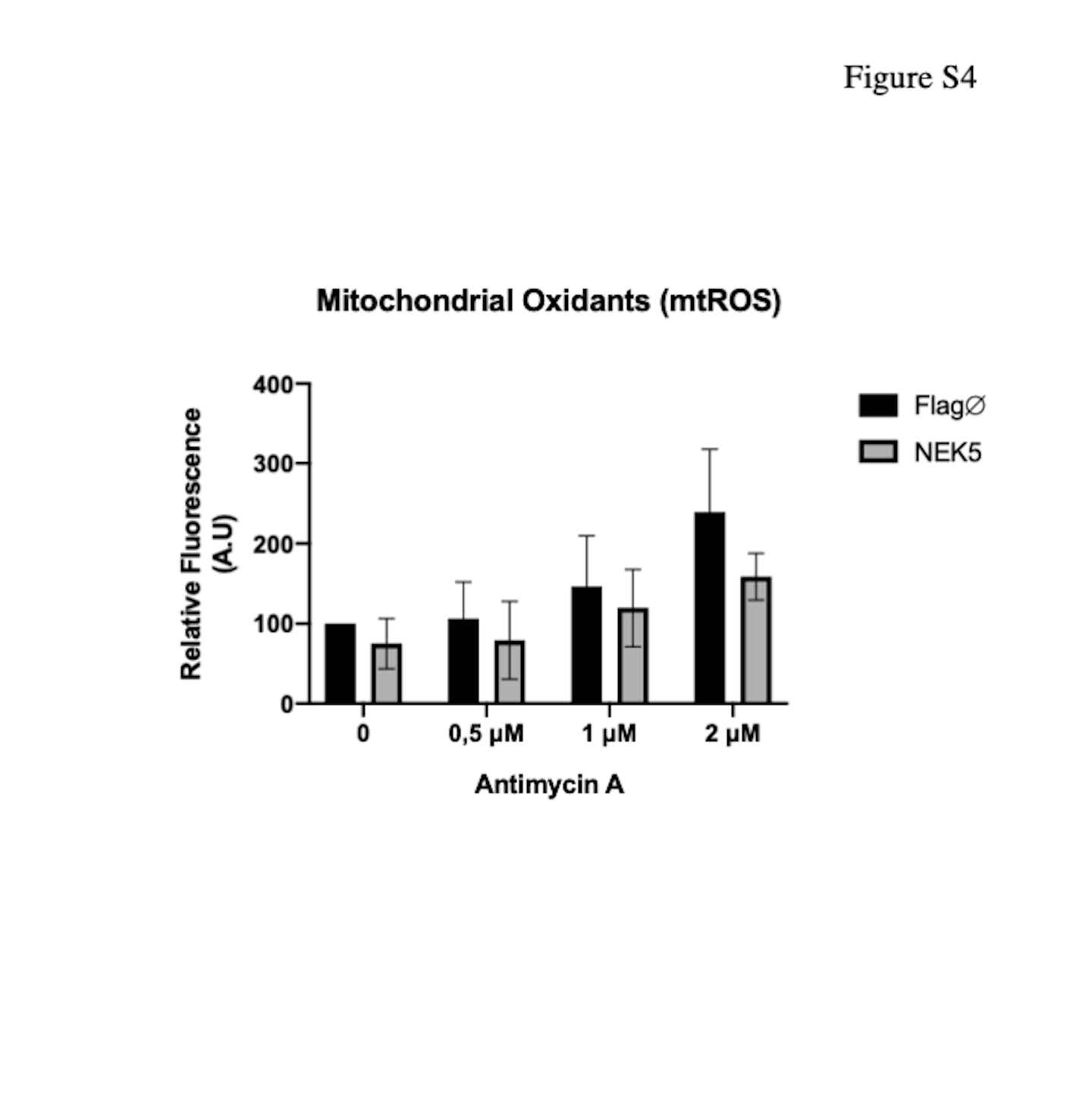

Supplement: Supplementary file 5 — Fig. S4. Overexpression of NEK5 does not significantly affects mitochondrial oxidants production. Flow Cytometry analysis of mitochondrial oxidants utilizing MitoSOX™ probe. The results showed no significant changes in mtROS suggesting that the increase in mtDNA integrity in NEK5WT are not related to mtROS. The average of three replicates is represented, and the bar indicates SD of n = 3. Student T‐test followed by Bonferroni post‐hoc was used as Statistical Test. [file FEB4-11-546-s005.tiff]
